# Metabolites and novel compounds with anti-microbial or antiaging activities from *Cordyceps fumosorosea*

**DOI:** 10.1186/s13568-022-01379-w

**Published:** 2022-04-02

**Authors:** Jie Wei, Xue Zhou, Mei Dong, Lufan Yang, Cheng Zhao, Ruili Lu, Guanhu Bao, Fenglin Hu

**Affiliations:** 1 Research Center on Entomogenous Fungi, Engineering Research Center of Fungal Biotechnology Ministry of Education, Anhui Agricultural University, West Changjiang Road No. 130, Hefei city, Hefei, 230036 Anhui Province China; 2 Natural Products Laboratory, State Key Laboratory of Tea Plant Biology and Utilization, Anhui Agricultural University, Hefei, 230036 Anhui Province China

**Keywords:** *Cordyceps fumosorosea*, Fumosoroseanosides, Fumosoroseain, Antibacterial, Antifungal, Antiaging

## Abstract

High-resolution mass spectrometry (HRMS) and nuclear magnetic resonance (NMR) analysis revealed that there are 20 main components in spores and mycelia extract of *Cordyceps fumosorosea* strain RCEF 6672 including mannitol (**1**), uridine (**2**), adenine (**3**). N^6^-(2-hydroxyethyl)-adenosine (**4**). N^6^-(2-hydroxyethylacetate)-adenosine (**5**), fumosoroseanoside A (**6**) and B (**7**), ovalicin-4α-alcohol (**8**), 1-linoleoyl-sn-glycero-3-phosphocholine (**9**) and its isomer (**10**), fumosoroseain A (**11**) and its isomer (**12**), 5 non-ribosomal peptides (**13** to **17**) and 3 fatty acids (**18** to **20**). The compounds **5**, **6**, **7**, **9** and **11** were prepared with preparative and semi-preparative HPLC and identified with 1D and 2D NMR. Compounds **4** and **5** were the first time identified from *C. fumosorosea*. Compounds **6**, **7** and **11** are novel compounds. Compounds **6** and **7** showed antibacterial and antifungal activities, and **11** showed antiaging activity. All the secondary metabolites (**4** to **8** and **11** to **17**) have strong bioactivities indicating that the metabolites have pharmaceutical development potentiality.

**Graphical Abstract:**

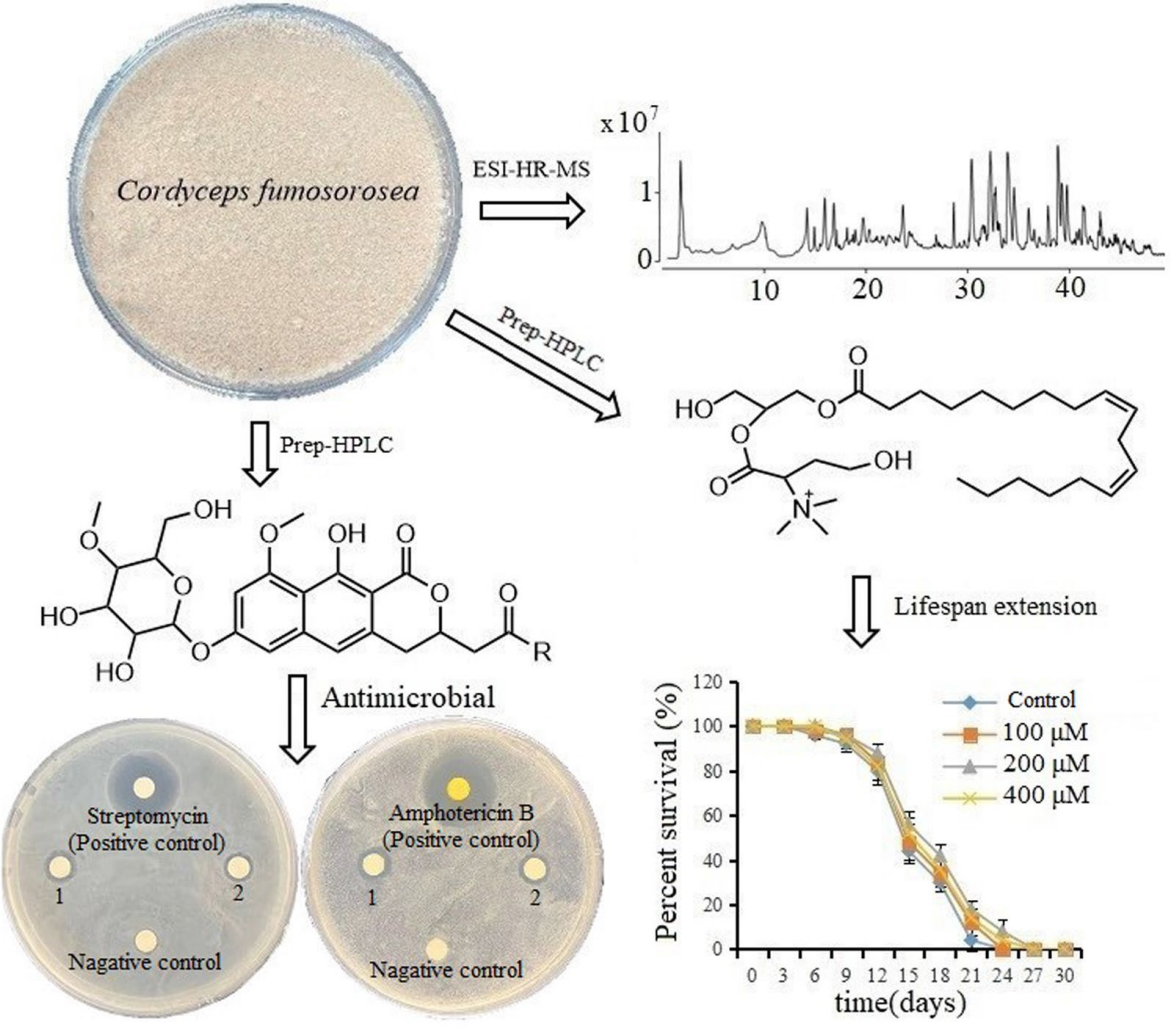

**Supplementary Information:**

The online version contains supplementary material available at 10.1186/s13568-022-01379-w.

## Keypoints

Clarified small molecular metabolites of *C. fumosorosea* for the first time.

Identified three novel compounds with antimicrobial or antiaging activities.

The fungus has development potentiality for rich in bioactive metabolites.

**Supplementary Information:**

The online version contains supplementary material available at 10.1186/s13568-022-01379-w.

## Introduction

*Cordyceps* are highly valued traditional medicines or healthy foods in Asian countries for a long history (Xiao et al. 2009; Liu et al. 2015, 2020; Lou et al. 2020). Some fungi of *Cordyceps* are also widely used in agriculture for pest biocontrol (Zhang et al. 2019; Kumar et al. 2021). Because long term coevolution with insects, the fungi of *Cordyceps* can synthesize a variety of bioactive metabolites such as cordycepin, militarinone, myriocin, cyclosporin, destruxins and enniatins etc. (Mularczyk et al. 2020; Hu and Li 2007; Isaka et al. 2000; Schmidt et al. 2002; Bagli et al. 1973; Wartburg and Traber 1988;Meca et al. 2010; Lu et al. 2013; Lu et al. 2014). As a well-known entomopathogenic fungi, *C. fumosorosea* can not only kill many kinds of pests but also synthesize more than 10 bioactive compounds, such as beauvericins, beauverolides, cepharosporolides, trichocaranes and fumosorinone A etc. (Hu and Li 2007; Hugo et al. 1983; Masahiko et al. 2005; Chen et al. 2018; Buchter et al. 2020). However, there is no metabolites reported from spores of the fungus yet. Meanwhile, our primary gene prediction based on the genome sequences (GCA-003025305.1) (Shang et al. 2016) with antiSMASH revealed that there were 40 secondary metabolic gene clusters in *C. fumosorosea*, including 28 non-ribosomal peptides (NRPs), 3 poly-ketone, 3 terpene, 1 siderophore, 5 NRPs or poly-ketone gene clusters. Our primary HPLC-HRMS analysis showed that *C. fumosorosea* had complicated metabolites including several possible unknown compounds. To make clear the structure and bioactivity of the unknown metabolites will be able to not only reveal the utilization value of the fungus, but also help us to evaluate chemical ecological safety of the fungal pesticide. Therefore, we launched a systematic analysis and identification of the metabolites of *C. fumosorosea*. In this study we will focus on the unknown metabolites. Through HPLC-HRMS guided isolation we can prepare the unknown compounds directly without the disturbance of the known metabolites. Because that *Cordyceps* do not rot easily in the soil and are usually used as tonic medicine or food, they are presumed to contain antibiotics and antiaging compounds (Ji et al. 2008; Schmidt et al. 2003; Olatunji et al. 2018; He et al. 2018), therefore, the isolated novel compounds of this study will be submitted to antimicrobial and antiaging tests. In this study *Escherichia coli* and *Candida albicans* will be used to test the antimicrobial activities, and *Caenorhabditis elegans* will be used for the antiaging assays. Nematode is widely used to study the biological effects of aging and human diseases because that its genome contains more than 18, 000 genes, of which 60 to 80% are homologous to human genes, meanwhile, it has highly conserved metabolic pathways and easy-to-maintain (Moliner et al. 2018; Calvo et al. 2016; Gruber et al. 2009).

## Materials and methods

### Chemicals

All solvents used for extraction were of analytical grade (Sinopharm Chemical Reagent Co., Ltd., Shanghai, China). HPLC grade methanol and formic acid were from Tedia Company of China (Shanghai, China). Deuterated NMR solvents were purchased from Cambridge Isotope Laboratory (Andover, MA, USA).

### Instrumentation

Preparative HPLC was performed using Agilent modules consisting of an autosampler PS410, two pumps M400, a UV detector 1260VWD and an automatic fraction collector 440FC. Semi-preparative HPLC was performed using Shimadzu modules consisting of an autosampler SIL-20A, two pumps LC-20AD and a PDA detector SPD-M20A. ESI-HR-MS data were obtained using an Agilent 1100 HPLC tandem 6510QTOF MS spectrometer. NMR experiments were recorded using a Bruker Advance 600 MHz spectrometer. Chemical shifts were referenced to the residual (CD_3_)_2_SO signal (δ_H_ 2.50, 3.33, δC 40.80) or CD_3_OD signal (δ_H_ 4.87, 3.31, δ_C_ 49.15) as internal standards for 1D NMR and 2D NMR.

### Fungal material

The fungal strain was isolated from *C. fumosorosea* infected lepidopterous larva collected in Anhui Province (China) and grown at 25 °C on PDA plates. The isolated strain was identified by morphology and sequence analysis of the ITS region of the rDNA. It has been catalogued RCEF 6672 and deposited in the Research Center for Entomogenous Fungi (RCEF, WDCM1031), Anhui Agricultural University.

### Fermentation, extraction and isolation

After 7 days cultivation on PDA plate, the spores of the fungus were harvested with a 0.05% Tween 80 solution to make a spore suspension (6.8 × 10^6^). 300 μL of the suspension was inoculated on a 150 mm PDA plate, and 2000 of the plates were inoculated for mass cultivation. After 10 days cultivation the spores and surface mycelia were scraped off the plates and freeze dried. The dried spores and mycelia were extracted with methanol and ethyl acetate mixture (1:1) under 40 kHz ultrasonication for three times in 30 min, and the total solid–liquid ratio is 1: 4 (w/v). The extract was evaporated under reduced pressure, and 10.5 g pasty crude extract was obtained. With the assistance of 40 kHz ultrasonication, 8 g of crude extract was redissolved in 10 ml of methanol. The turbid liquid was centrifuged at 10,000 *g* for 10 min, and the supernatant was separated by preparative HPLC with an Agilent Prep-C18 (21.2 mm × 250 mm, 10 μm) column for primary isolation, and semi-preparative HPLC with an Agilent SB-C18 (9.4 mm × 250 mm, 5 μm) column for final purification. The conditions for preparative HPLC are as follows: injection volume 150 μL; flow rate 15 mL/min; elution gradient: 0–15 min, 30% to 100% methanol, 15–20 min, 100% methanol, 20—22 min, 100% to 30%. The effluents were collected every one minute for one tube and tested by HPLC-HRMS. The tube contained unknown compounds was condensed under reduced pressure and submitted to semi-preparative HPLC. The conditions for semi-preparative HPLC are as follows: injection volume 30 μL; flow rate 3 mL/min; elution gradient: 0–1 min, 40% methanol, 1–15 min, 40% to 100% methanol, 15–18 min, 100% methanol, 18–22 min, 100% to 40% methanol, 22–25 min, 40% methanol. The effluents of the unknown compounds were collected according to the m/z of the unknown compounds detected by HRMS through a 10:1 splitter.

### HPLC-HRMS analysis

The crude extract and prepared pure compounds were analyzed with an Agilent Poroshell 120 EC-C18 (2.7 μm, 3.0 × 100 mm) column, and the LC parameters were set as follows: injection volume, 5 μL; column temperature, 25 °C; and flow rate, 0.4 mL/min. The mobile phase was composed of (A) 0.1% formic acid in water and (B) 0.1% formic acid in acetonitrile, and a gradient elution was carried out: 0–40 min, 5–100% B, 40–45 min, 100% B, 45–55 min, 100–5% B. The eluates were monitored with a PDA performing a full wavelength scan from 200 to 600 nm, and a TOF MS with following parameter settings: gas temperature, 350 °C; drying gas, 10 L/min; nebulizer pressure, 45 psi; capillary voltage, 4000 V in positive mode and 3500 V in negative mode; fragmentor voltage, 215 V in positive mode and 170 V in negative mode; skimmer voltage, 60 V. Data acquisition was performed in the m/z range of 50–1700 Da. The effluents of the preparative HPLC were detected by HPLC-HRMS without chromatographic column.

### Metabolite identification

The molecular formulas of the metabolites were calculated by Mass Hunter based on accurate mass and isotopic pattern recognition. Compounds were putatively identified by searching the molecular formulas against the in-house entomopathogenic fungi database and the *Dictionary of Natural Products* (http://dnp.chemnetbase.com/). The known compounds were confirmed by UV/visible spectra whenever possible, and verified by their elution order (polarity) and structure characteristics. Some common metabolites of entomopathogenic fungi such as mannitol, adenosine and beauvericin A were confirmed with standards. Molecular formulas without corresponding compounds in the database were labeled as unknown compounds and submitted to identification with 1D and 2D NMR.

### Antimicrobial activity

The antimicrobial activities of the metabolites were evaluated by the disc diffusion assay according to the method reported by Wu et al. (Wu et al. 2018). Streptomycin was used as positive control for antibacterial tests against *E. coli* (ATCC 25922). Amphotericin B was used as positive control for the antifungal bioassays against *C. albicans* strain CMCC98001 (from China General Microbiological Culture Collection Center). All samples were prepared to a final concentration of 1, 2 and 3 mg/mL in 1.5% DMSO, and 10 μL were loaded on the paper discs (6.0 diameters). The width of the transparent antimicrobial zone was measured with a vernier caliper. Each experiment was repeated three times.

### Determination of Antiaging activity

Antiaging activity tests included lifespan test, reproduction assay, locomotion assay and thermotolerance assay. *C. elegans* (bought from Caenorhabditis Genetics Center, University of Minnesota) was fed with *E. coli* strain OP50 according to the method reported by Onken and Driscoll (2010). The nematodes were treated with a solution of sodium hypochlorite and sodium hydroxide to make the nematode eggs hatching and growing synchronously according to the reported method (Rathor et al. 2017).

Lifespan was measured according to the method of Liu et al. (2016). The synchronized L4 larvae (3 days old, 50 per plate) were cultivated on NGM plate (containing *E. coli* strain OP50) at 20 °C with different content of compounds (100, 200, 400 μM) or 0.1% DMSO as control. The nematodes were transferred to new plates every two days to ensure adequate food, and the number of dead worms were recoded simultaneously.

Reproduction assay was carried out according to the method reported by Chen et al. (2014). Each plate contained only one L4 larva which was transferred to fresh plate every 24 h until the nematode no longer laid eggs. The number of offspring produced by each nematode in the whole breeding period were calculated and summed to obtain the total number of offspring.

Locomotion assay was performed according to the method of Wilson et al. (2006). 50 *C. elegans* were cultivated and treated with compounds as described in lifespan assays. On the 5th, 8th, 12th and 16th day of adulthood the nematodes were counted and classified into three classes: motion A nematodes moved constantly, motion B nematodes only moved when prodded and motion C nematodes sway only head or tail if prodded.

Thermotolerance assay was performed according to the Wilson’s method (Wilson et al. 2006). 50 nematodes were cultivated and treated with compounds as described in lifespan assays. After four days of culture, the nematodes were exposed to 35 °C, and the dead nematodes were monitored every 1 h until all the nematodes died.

## Results

### Primary analysis of the main metabolites of *C. fumosorosea*

**Fig. 1:**
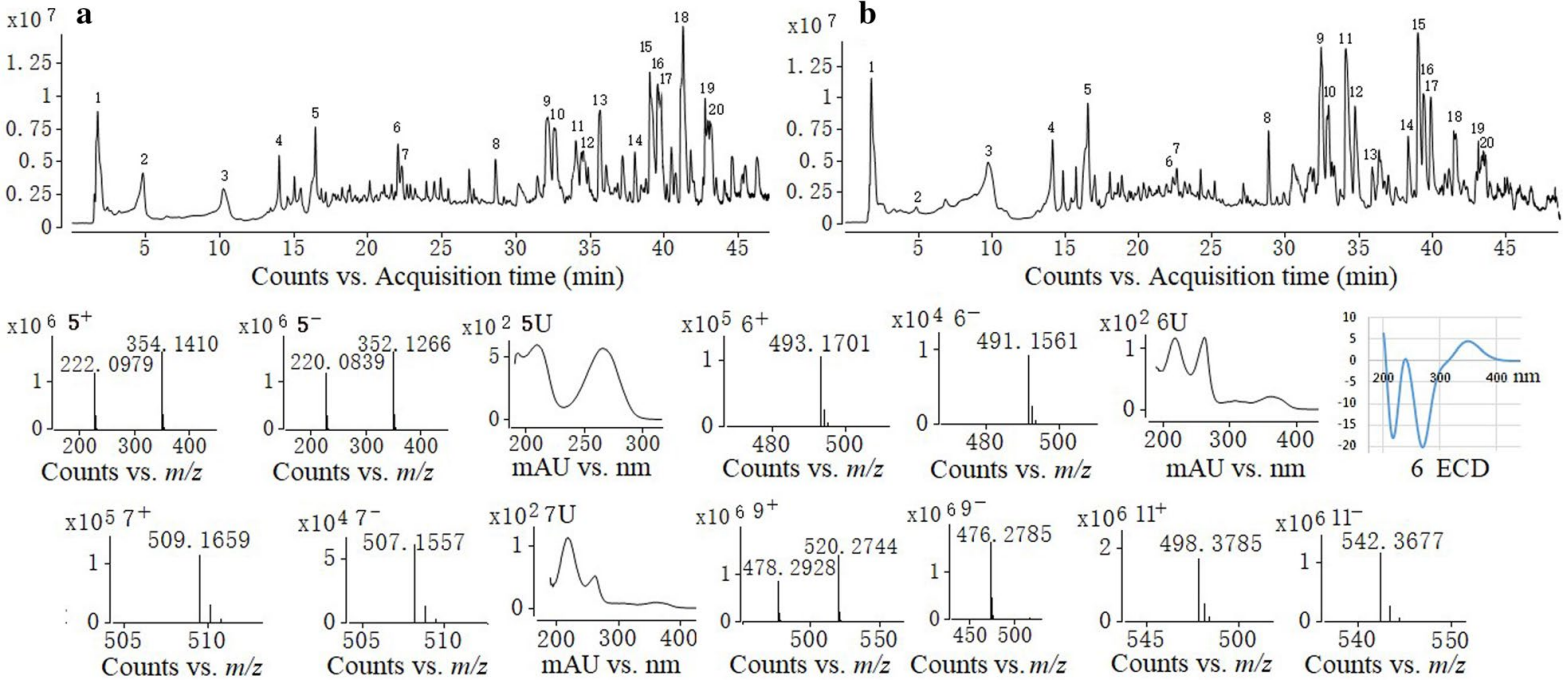
HPLC-HRMS analysis of the extract form spores and mycelia of *Cordyceps fumosorosea. ***A** is the base peak chromatogram of the crude extract in positive model; **B** is the base peak chromatogram of the crude extract in negative model; number 5, 6, 7, 9 and 11 are the five possible unknown metabolites, and ‘ + ’, ‘ − ’, ‘U’ and ‘ECD’ mean cation, anion, ultraviolet spectrum and circular dichroism of the metabolites respectively

HPLC-HRMS analysis showed that there are 20 main metabolites in the extract from mycelia and spores of *C. fumosorosea* (Fig. 1). Database query according to the high-resolution (HR) mass and UV spectra (Fig. 1) and standards comparison found there were 13 known compounds (Abraham et al. 2015; Daniela et al. 2020; Manrico et al. 1975), and 7 possible unknown compounds, which were not found in the *Dictionary of Natural Products* database (Table 1).

**Table 1:** Main metabolites of extract form spores and mycelia of *Cordyceps fumosorosea*

Retention time (min) (Number)	Mass (m/z) and erro (mDa)	Uvλmax (nm)	Molecular formula	Name
1.66 (1)	[M−H]^−^181.0729, 1.1	200	C_6_H_14_O_6_	Mannitol^a^
4.83 (2)	[M−H]^−^ 243.0625, 0.2; [2 M−H]^−^ 487.1321, 0.3 [M + H]^+^ 245.0763, 0.5; [M-C_5_H_7_O_4_]^+^113.0349, 0.3	206, 261	C_9_H_12_N_2_O_6_	Uridine^a^
9.74 (3)	[M-H]^−^ 266.0899, 0.4; [M-C_5_H_9_O_4_]^−^ 134.0476, 0.4 [M + H]^+^ 268.1045, 0.5; [M-C_5_H_7_O_4_]^+^136.0822, 0.6	205, 258	C_10_H_13_N_5_O_4_	Adenine^a^
14.13 (4)	[M−H]^−^ 310.1155, 0.2; [M-C_5_H_9_O_4_]^−^ 178.0731, 0.3 [M + H]^+^ 312.1305, 0.3; [M-C_5_H_7_O_4_]^+^180.0876, 0.4	209, 265	C_12_H_17_N_5_O_5_	N^6^-(2-Hydroxyethyl)-Adenosine^a^
16.41 (5)	[M−H]^−^ 352.1266, 0.3; [M-C_5_H_9_O_4_]^−^ 220.0839, 0.1 [M + H]^+^ 354.1410, 0.2; [M-C_5_H_7_O_4_]^+^ 222.0979, 0.7	209, 265	C_14_H_19_N_5_O_6_	Unknown
22.24 (6)	[M−H]^−^ 491.1565, 0.6; [M + H]^+^ 493.1699, 0.5	262, 361	C_24_H_28_O_11_	Unknown
22.43 (7)	[M−H]^−^ 507.1513, 0.5; [M + H]^+^ 509.1658, 0.4	262, 361	C_24_H_28_O_12_	Unknown
28.85 (8)	[M−H]^−^ 297.1718, 1.1; [M + H]^+^ 299.1859, 0.6; [M + Na]^+^ 321.1680, 0.8	200	C_16_H_26_O_5_	Ovalicin-4α-alcohol
32.41 (9) 32.88 (10)	[M−H]^−^ 476.2785, 0.2; [M + H]^+^ 478.2928, 0; [M + Na]^+^ 500.2744, 0.4	200	C_23_H_44_NO_7_P	Unknown
34.28 (11) 34.83 (12)	[M + COOH]^−^ 542.3695, 0.3; [M + H]^+^ 498.3791, 0.2; [M-C_18_H_29_O]^+^ 236.1495, 0.3	200	C_28_H_51_NO_6_	Unknown
35.94 (13)	[M−H]^−^ 486.2863, 0.5; [M + H]^+^ 488.3010, 0.7	215	C_27_H_41_N_3_O_5_	Beauveriolide I^b^
38.36 (14)	[M−H]^−^ 734.4031, 0.9; [M + H]^+^ 736.4173, 0.5; [M + NH_4_]^+^ 753.4438, 0.5; [M + Na]^+^ 758.3985, 0.2	210	C_41_H_57_N_3_O_9_	Beauveriolide E
39.06 (15)	[M-H]^−^ 782.4039, 0.7; [M + H]^+^ 784.4171, 0.3; [M + NH_4_]^+^ 801.4439, 0.6; [M + Na]^+^ 806.3991, 0.4	255	C_45_H_57_N_3_O_9_	Beauvericin^b^
39.45 (16)	[M-H]^−^ 804.3469, 0.8; [M + H]^+^ 806.3612, 0.5	225, 280	C_41_H_51_N_5_O_12_	Conoideocrellide D
39.92 (17)	[M−H]^−^ 796.4185, 0.6; [M + H]^+^ 798.4331, 0.7; [M + NH_4_]^+^ 815.465, 0.6; [M + Na]^+^ 820.4138, 0.8	260	C_46_H_59_N_3_O_9_	Beauvericin A^a^
41.69 (18)	[M−H]^−^ 279.2332, 0.2; [M + H]^+^ 281.2469, 0.6	200	C_18_H_32_O_2_	Linoleic acid
43.21 (19)	[M−H]^−^ 255.2334, 0.4; [M + H]^+^ 257.2473, 0.2	200	C_16_H_32_O_2_	Palmitic acid
43.54 (20)	[M−H]^−^ 281.2481, 0.5; [M + H]^+^ 283.2629, 0.3	200	C_18_H_34_O_2_	Oleic acid

^a^Confirmed with standards

^b^Have been reported from *Cordyceps fumosorosea*, *Isaria fumosorosea* or *Paecilomyces fumosoroseus* ^33–35^

Among the 7 possible unknown compounds the **9** and **10**, **11** and **12** have the same mass and ultraviolet spectrum respectively, indicating that they are two pairs of stereoisomers. Five of the unknown compounds were prepared by HRMS guided isolation with preparative HPLC and semi-preparative HPLC according to the above-mentioned methods. We obtained 11.3 mg compound **5**, 17.6 mg compound **6**, 5.3 mg compound **7**, 9.5 mg compound **9** and 16.9 mg compound **11**. Compound **5** is white powder. Compounds **6** and **7** are light-yellow amorphous powder, and the compound **9** and **11** are colorless oil. The purity and molecular formulas of the prepared compounds was confirmed by HPLC-HRMS (Fig. 1) and submitted to NMR analysis.

### Structure elucidation of the isolated compounds

**Fig. 2:**
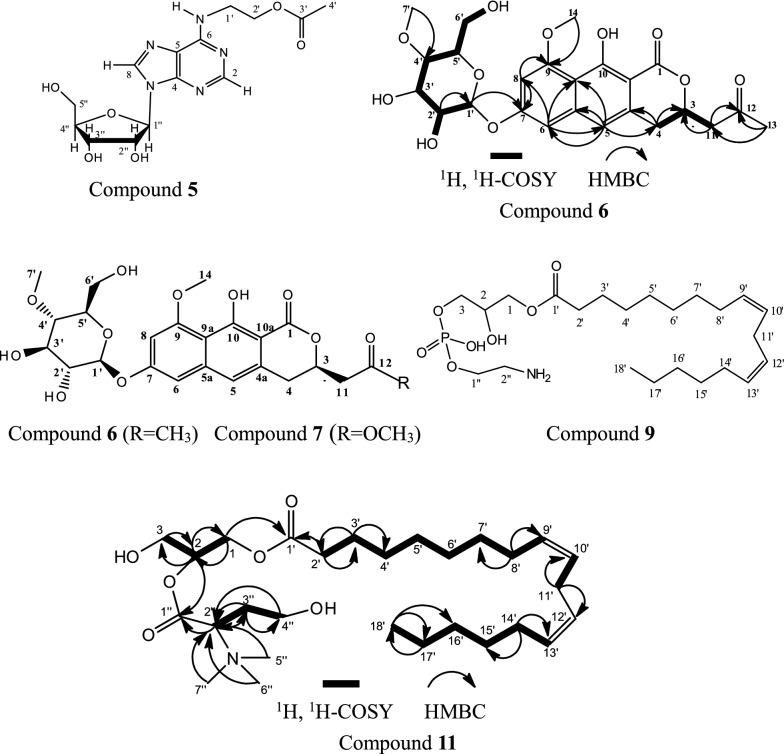
Structures of compounds **5**, **6**, **7**, **9** and **11**, and HMBC and ^1^H-^1^H COSY correlations of the compounds **6**, **7** and **11**

Compound **5**: The molecular formula was calculated as C_14_H_19_N_5_O_6_ based on its ESI-HRMS (Fig. 1). The characteristic UV absorptions (λ_max_) are at 209 and 265 nm (Fig. 1). The ^1^H- and ^13^C NMR spectra of **5** (Additional file 1: Fig. S1) showed characteristic signals for adenosine, a major constituent of Cordyceps (Hu and Li 2007). There are two methylene signals at δ_H_ 3.84 (2H, m) and 4.27 (2H, t, J = 5.4 Hz) and δc 39.1 and 62.7, which were very similar to those of N^6^-(2-hydroxyethyl) adenosine (Furuya et al. 1983). However, additional signals for an acetyl moiety were observed at δ_H_ 2.05 (3H, s), δ_C_ 19.4 and 171.5 in the HMBC spectrum, which suggested **5** as an acetylated form of N^6^ -(2-hydroxyethyl) adenosine. The position of an acetyl moiety was determined from the HMBC correlation between δ_C_ 171.5 and δ_H_ 4.27 (H-2ʹ). Taken together, the structure of **5** was determined as N^6^-(2-hydroxyethylacetate) adenosine (Fig. 2). The NMR signals assignment was list in Table 2. Literature inquiry showed that N^6^-(2-hydroxyethylacetate) adenosine is the known compound cordyrrole B which can significantly inhibit adipocyte differentiation and pancreatic lipase (Kim et al. 2014).

**Table 2:** 1D and 2D NMR spectral data (δ in ppm) of compound **5**

Position	^13^C (δ)	DEPT, HMQC	δ_H_, mult (J in Hz)	^1^H,^1^H-COSY (δ)	HMBC (δ)
2	152.1	CH	8.19, br		
4	148.0	C			
5	120.2	C			
6	155.0	C			
8	140.3	CH	8.23, s		
1′	39.1	CH2	3.84, m	4.27	
2′	62.7	CH2	4.27, t (5.4)		
3′	171.5	C			
4′	19.4	CH3	2.00, s		171.5
1″	89.9	CH	5.93, d (6.4)	4.71	74.1, 140.3, 148.0
2″	74.1	CH	4.71, dd (6.4, 5.1)	4.30, 5.93	89.9
3″	71.3	CH	4.30, dd (5.1, 2.7)	4.71, 4.14	62.2, 89.9
4″	86.9	CH	4.14, q (2.6)	3.71, 3.85, 4.30	71.3
5″	62.2	CH2	3.71, dd (12.5, 2.7); 3.85 dd (12.5, 2.7)	3.85, 4.14; 3.71, 4.14	71.3, 86.9

Compound **6**: The molecular formula was calculated as C_24_H_28_O_11_ based on its ESI-HRMS (Fig. 1). The characteristic UV absorptions (λ_max_) are at 260, 310, and 360 nm (Fig. 1). ^13^C and DEPT135 (Additional file 1: Fig. S2) showed that compound **6** has 24 carbon atoms including 9 quaternary carbons, 9 tertiary carbons, 3 secondary carbons and 3 primary carbons which connected 24 hydrogen atoms. The other 4 hydrogens in the molecule are probably active hydrogens. The chemical shifts of ^1^H and ^13^C and coupling of C–H obtained by 1D and 2D NMR (Fig. 2; Additional file 1: Fig S1) spectra are shown in Table 3. The carbons at δ101.2, δ79.4, δ76.5, δ76.2, δ74.0, δ60.6 and δ60.0, and coupling relationships between the hydrogens connected to the carbons (Fig. 2) according well with a 4′-methoxyl-glucose moiety which existed commonly in metabolites of entomopathogenic fungus (Hu et al. 2002, 2009). The chemical shifts and coupling relationships between aromatic carbons and hydrogens (Fig. 2) showed that the aglycone is a derivative of 1H-naphtho[2,3-c] pyran-1-ones which is consistent with the report (Ayer et al. 1991). The chemical shift at 205.5 indicated the existence of ketone. The long-range coupling between the hydrogens of the two methoxy groups and carbons at δ 79.4 and 161.6 (Fig. 2) confirmed that the methoxy groups are at the position of Glu-C4' and the position 9 respectively. The terminal hydrogen at δ 4.99 has a double peak on the ^1^H spectrum with 7.2 Hz coupling constant, indicating that the glucosyl group is β configuration. The ECD spectrum (Fig. 1) showed the compound is a R configuration. Based on above analysis the structure of the new compound (**6**) was depicted in Fig. 2 and named as fumosoroseanoside A.

**Table 3:** 1D and 2D NMR spectral data (δ in ppm) of compound **6**, and ^1^H and ^13^C NMR spectral data of compound **7**

Compound **6**						Compound 7	
Position	^13^C (δ)	DEPT135, HMQC	δ_H_, mult (J in Hz)	^1^H,^1^H-COSY (δ)	HMBC (δ)	^13^C (δ)	δ_H_, mult (J in Hz)
1	170.4	C				170.5	
3	75.3	CH	4.97, m	2.96, 3.04; 2.91, 2.98		75.1	4.86, m
4	32.6	CH_2_	2.96, m; 3.04, m	2.96, 3.04, 4.97	134.7	33.1	3.00, m; 3.03, m
4a	134.7	C				134.6	
5	115.6	CH	6.97, s		100.9, 110.3, 32.6, 140.9	115.5	6.88, s
5a	140.9	C				140.9	
6	100.9	CH	6.84, d (2.3)	6.74	110.3, 115.6, 102.0, 161.6	100.9	6.82, d (2.3)
7	158.1	C				158.1	
8	102	CH	6.74, d(2.2)	6.84	110.3, 161.6, 158.1, 101.2	102	6.72, d (2.2)
9	161.6	C				161.6	
9a	110.3	C				110.3	
10	163.4	C				163.4	
10a	101.3	C				101.3	
11	47.7	CH_2_	2.91, dd(17.1, 5.2); 2.98, dd (17.2, 7.3)	2.91, 2.98, 4.97	205.5, 75.3	40.6	2.79, dd (16.1, 8.1), 2.88, dd (16.1, 4.3)
12	205.5	C				170.4	
13(R)	30.6	CH_3_	2.14, s		205.5, 47.7	52.2	3.63, s
14	56.0	CH_3_	3.83, s			56.1	3.80, s
1′	101.2	CH	4.99, d (7.2)	3.38	158.1	101.2	4.99, d (7.2)
2′	74	CH	3.38, m	4.99, 5.23, 3.42	101.2	74	3.38, m
3′	76.2	CH	3.42, m	3.02, 3.38		76.2	3.42, m
4′	79.4	CH	3.02, m	3.41, 3.42	76.2, 76.5	79.4	3.02, m
5′	76.5	CH	3.41, m	3.02		76.5	3.41, m
6′	60.6	CH_2_	3.48, m; 3.62, m	3.48, 3.62, 3.42, 4.71		60.6	3.48, m; 3.62, m
7′	60.0	CH_3_	3.43, s		79.4	60.0	3.43, s
		OH	3.3				
		OH	4.71	3.48, 3.62			
		OH	5.23	3.38			
		OH	8.35				

Compound **7**: The molecular formula was assigned as C_24_H_28_O_12_ based on its ESI-HRMS (Fig. 1). Compound **7** has just one more oxygen atom than compound **6**, and they have the same UV absorptions (Additional file 1: Fig. S2). Obviously, compound **7** is possibly an oxo-derivate of fumosoroseanoside A, and the extra oxygen atom is not in the conjugated system of the molecule. The ^1^H and ^13^C NMR spectra (Additional file 1: Fig. S2) of compound **7** are shown in Table 3. By comparing the ^1^H and ^13^C NMR between compound **7** and compound **6**, the methyl signals of compound 7 (δ_H_ 2.14, δ_C_ 30.6) were disappeared, while one more methoxy group (δ_H_ 3.63, δ_C_ 52.0) appeared. Meanwhile, the keto carbon at δ_C_ 205.5 shifted to a higher field δ_C_ 170.5. Obviously, the methyl group in compound **6** which combined to the carbonyl carbon of the ketone group was oxidized and formed a methyl ester group. Based on above analysis the structure of the new compound (**7**) was shown in Fig. 2 and named as fumosoroseanoside B.

Compound **9**: The molecular formula was calculated as C_23_H_44_NO_7_P based on its ESI-HRMS (Fig. 1). ^31^P-NMR confirmed the existence of phosphorus atom. ^13^C and DEPT (Additional file 1: Fig. S4) showed a typical linoleic acid group with a carbonyl carbon (δ_C_ 173.7), 4 unsaturated carbons (δ_C_ 129.07, 129.13, 130. 90, 130.97), 12 methylene carbon (δ_C_ 23 to 34) and a methyl carbon (δ_C_ 14.4). The chemical shifts of the other 5 carbons including 4 methylenes (δ_C_ 63.2, 66.2, 67.8, 41.8) and 1 methine carbons (δ_C_ 69.9) suggested the existence of a glycerol and an ethanolamine group. Carbons δ_C_ 69.9, 67.8, 63.2 and 41.8 were split with J = 7.5, 5.7, 6.6 and 5.2 Hz respectively suggesting that they had two or three bonds coupling with the phosphorus atom of phosphate group. The coupling constants between the phosphorus atom and the second carbon (J = 7.5) and the third carbon (J = 5.7) of the glycerol moiety suggested that the esterification position is the third carbon of the glycerol group. Therefore, compound 9 is 1-linoleoyl-sn-glycero-3-phosphocholine (Fig. 2). The structure was confirmed with ^1^H, COSY, HMQC and HMBC (Additional file 1: Fig. S4) and the signals assignment was list in Table 4. Despite that this compound has been reported several times (He et al. 2018; Luo et al. 2013) it is the first time identified with one- and two-dimension NMR from microorganism.

**Table 4:** 1D and 2D NMR spectral data (δ in ppm) of compound **9**

Position	^13^C (δ)	DEPT, HMQC	δ_H_, mult (J in Hz)	COSY (δ)	HMBC (δ)
1	66.23	CH_2_	4.13, dd (11.3, 6.1);	4.20; 4.00	175.41, 69.9
			4.20, dd (11.3, 4.6)	4.13; 4.00	
2	69.90 (d, J = 7.5)	CH	4.00, quint (5.3)	3.92	67.80, 66.23
3	67.80 (d, J = 5.7)	CH_2_	3.92, m	4.0	68.54
1′	175.41	C			
2′	34.93	CH_2_	2.38, t (7.5)	1.65	26.0
3′	26.0	CH_2_	1.65, m	2.38; 1.36	34.93
4′	30.23	CH_2_	1.34–1.37	1.65	26.0–30.48
5′	30.24	CH_2_			
6′	30.32	CH_2_			
7′	30.48	CH_2_			
8′	28.17	CH_2_	2.09, dd (13.17, 6.37)	1.39; 5.39	130.87
9′	130.87	CH	5.39, m	2.09	28.17
10′	129.13	CH	5.33, m	2.80	26.56
11′	26.56	CH_2_	2.80, t (6.3)	5.33	129.07, 129.13
12′	129.07	CH	5.33, m	2.76	25.56
13′	130.96	CH	5.39, m	2.09	27.17
14′	28.17	CH_2_	2.09, dd (13.17, 6.37)	1.39; 5.39	130.96
15′	30.74	CH_2_	1.34–1.37		
16′	32.64	CH_2_			
17′	23.60	CH_2_	1.33, m	0.93	14.45
18′	14.45	CH_3_	0.93, t (6.7)	1.33	23.60
1″	63.23 (d, J = 5.2)	CH_2_	4.08, dt (7.4, 5.0)	3.17	41.78
2″	41.78 (d, J = 6.6)	CH_2_	3.17, t (5.0)	4.08	63.23

Compound **11**: The molecular formula was assigned as C_28_H_52_NO_6_ based on its ESI-HRMS (Fig. 1). The UV absorptions (λ_max_) are at 231 nm (Fig. 1). ^13^C, DEPT90 and DEPT135 (Additional file 1: Fig. S5) showed a typical linoleic acid group with a carbonyl carbon (δ_C_ 174.1), 4 unsaturated carbons (δ_C_ 129.96, 129.87, 128.13, 128.06), 12 methylene carbon (δ_C_ 25 to 34) and a methyl carbon (δ_C_ 13.48). The three methyl groups with the same chemical shift values (δ_C_ 51.41, δ_H_ 3.20) are consistent with the characteristics of trimethylamine. Long-range coupling between the hydrogen (δ_H_ 3.75) of the trimethylamine connected methine and the carbonyl carbon (δ_C_ 170.75) and an ethanol group (Fig. 2) suggested the existence of a 2-trimethylamino-4-hydroxybutyrate moieties. Long-range coupling between the glycerol hydrogen and δ_C_ 174.10, δ_C_ 170.75 revealed that the linoleic acid and 2-trimethylamino-4-hydroxybutyrate moieties were connected to C_1_ and C_2_ of the glycerol moiety, respectively. Therefore, compound **11** is 1-linoleoyl-sn-glycero-2-(2-trimethylamino-4-hydroxybutyric ester). Its structure and hydrocarbon attributes are shown in Fig. 2 and Table 5. This compound was found to be a new compound by database and literatures enquiry and named as fumosoroseain A.

**Table 5:** 1D and 2D NMR spectral data (δ in ppm) of compound **11**

Position	^13^C (δ)	DEPT, HMQC	δ_H_, mult (J in Hz)	COSY (δ)	HMBC (δ)
1	65.52	CH_2_	4.06, dd (11.5, 6.0);	4.15; 3.94	68.54; 174.31
			4.15, dd (11.5, 4.7)	4.06; 3.94	
2	68.54	CH	3.94, quint (5.4)	3.46; 3.51	65.52; 170.75
3	72.35	CH_2_	3.46, dd (10.5, 4.6)	3.51; 3.94	68.54
			3.51, dd (10.5, 5.6)	3.46; 3.94	
1′	174.31	C			
2′	33.94	CH_2_	2.34, t (7.5)	1.61	25.0; 174.31
3′	25.0	CH_2_	1.61, m	2.34; 1.32	33.94; 29.70
4′	29.16	CH_2_	1.30–1.35	1.35–1.29; 1.61; 2.05	25.0–29.70
5′	29.19	CH_2_			
6′	29.29	CH_2_			
7′	29.7	CH_2_			
8′	27.17	CH_2_	2.05, m	1.35; 5.35	129.87/129.96; 29.16/29.19
9′	129.96	CH	5.35, m	2.05	27.17
10′	128.13	CH	5.31, m	2.76	25.56
11′	25.56	CH_2_	2.76, t (6.6)	5.31	128.06/128.13
12′	128.06	CH	5.31, m	2.76	25.56
13′	129.87	CH	5.35, m	2.05	27.17
14′	27.17	CH_2_	2.05, m	1.35; 5.35	129.87/129.96; 29.16/29.19
15′	29.46	CH_2_	1.30–1.35	1.35–1.29; 1.61; 2.05	25.0–29.70
16′	31.64	CH_2_			
17′	22.6	CH_2_	1.31, m	0.9	13.48
18′	13.48	CH_3_	0.90, t (6.5)	1.31	22.60; 31.64
1″	170.75	C			
2″	76.67	CH	3.75, dd (11.2, 2.6)	2.07	170.75; 28.03; 51.04
3″	28.03	CH_2_	2.07, m;	2.23; 3.75	76.67; 67.55
			2.23, m	2.07; 3.55; 3.65	
4″	67.55	CH_2_	3.55, m;	3.65; 2.23; 2.07	76.67
			3.65, m	3.55; 2.23; 2.07	
5–7″	51.41	3CH_3_	3.20, s		76.67

### Bioactivities determination

Bioactivities of N^6^-(2-hydroxyethylacetate) adenosine and linoleoyl-glycero-phosphocholine have been reported already (Kim et al. 2014; Harrison et al. 2020; Papandreou et al. 2021; Azarcoya-Barrera et al. 2021), therefore we just submitted the 3 novel compounds for bioactivity studies. Because *Cordyceps* are presumed to contain antibiotics and antiaging compounds (Ji et al. 2008; Schmidt et al. 2003; Olatunji et al. 2018; He et al. 2018), the isolated novel compounds were submitted to antimicrobial and antiaging tests.

### Antimicrobial activity

**Fig. 3:**
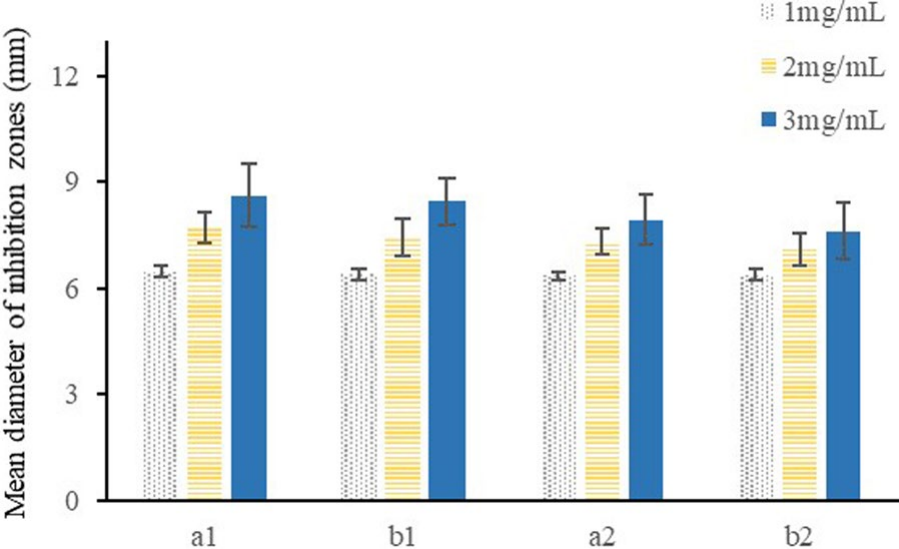
Antibacterial and antifungal activities of compounds **6** and **7**. The mean diameter of inhibition zones resulting from the antimicrobial activities of compounds 6 and 7. **a1** Results of compound 6 against *C. albicans*. **b1** Results of compound 6 against *E. coli.* **a2** Results of compound 7 against *C. albicans*. **b2** Results of compound 7 against *E. coli*

Compound **6** and **7** showed significant (*P* ˂ 0.05) antibacterial and antifungal activities compared with the negative control (the solvent) at the experimental doses from 10 to 30 μg per paper disk (Fig. 3). Compound **6** had slightly stronger inhibition ability on microbial than compound **7**, while compound **11** did not show detectable inhibitory effect against bacteria or fungi at the experimental doses.

### Antiaging activity

Because compound **6** and **7** can inhibit *E. coli* and make nematodes starve to death, only compound **11** was submitted to nematodes antiaging model.

**Fig. 4:**
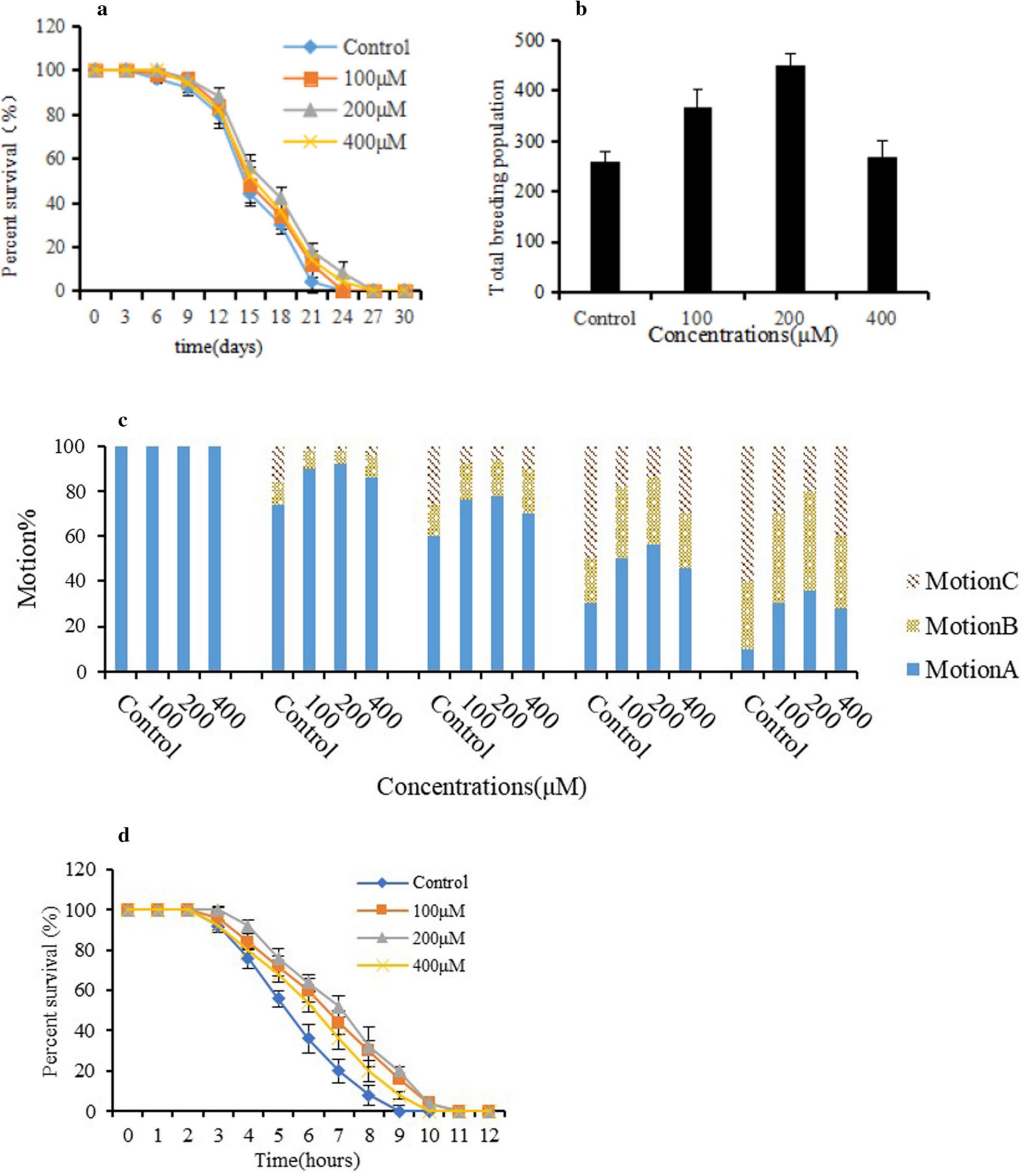
Antiaging activities of compound **11.** **a** Effect of compound 11 on lifespan of *C. elegans* (Control, equal volume solvent). **b** Effect of compound 11 on reproduction of *C. elegans* (Control, equal volume solvent). **c** Effect of compound 11 on locomotion (Control, equal volume solvent). **d** Effect of compound 11 on heat stress tolerance of *C. elegans* (Control, equal volume solvent)

Lifespan was the main antiaging indicator of *C. elegans*. As shown in Fig. 4, compound **11** could extend the lifespan of the nematodes at the concentration from 100 μM to 400 μM, and at 200 μM the compound has the most significant activity (*P* < 0.05), and the average lifespan was extended by 11.3% at 200 μM (Fig. 4a). Results of the reproduction tests (Fig. 4b) showed that the number of eggs per day of 100 μg and 200 μg treatment group were 368 ± 35 and 450.61 ± 52.21, which is significantly higher than that of the control group (*P* < 0.05). But the 400 μM treatment group had no significant effect on promoting reproduction (*P* > 0.05). Results of the locomotion tests (Fig. 4c) showed that with the aging of the nematodes, the constantly moved nematodes (Motion A) gradually decreased, but the addition of compound **11** could significantly delay the decline of the nematode's motion ability (P < 0.05). In the heat shock experiment (Fig. 4d), the survival rate of nematodes treated with compound **11** was increased, especially after 5 h treatment, the survival rate of the treated group significantly higher than that of the control group (*P* < 0.05).

Results of above tests showed that compound **11** can significantly prolong lifespan, increase fertility and enhance stress tolerance. This indicated that compound **11** had significant antiaging activity at the tested concentrations. Compound **11** (fumosoroseain A) is a kind of lecithin derivative which usually possess many bioactivities including anti-aging bioactivity at a suitable concentration (Papandreou et al. 2021; Azarcoya-Barrera et al. 2021; Derbyshire and Obeid 2020; Zhang et al. 2021), but how the compound 11 plays anti-aging bioactivity needs to be further studied.

## Discussions

Above analysis showed that there are 20 main small molecular metabolites in mycelia and spores extract of *C. fumosorosea* including 8 primary metabolites and 12 secondary metabolites. The primary metabolites included 1 nucleobase (**2**), 1 nucleoside (**3**), 3 fatty acids (**18** to **20**) and 2 phospholipids (**9** and **10**). Among them adenosine (**3**) is one of the main healthy constituents of Cordyceps (Hu and Li 2007; He et al. 2018; Luo et al. 2013, and phospholipids (**9** and **10**) are nutrients with many bioactivities (Azarcoya-Barrera et al. 2021; Derbyshire and Obeid 2020; Zhang et al. 2021). Mannitol (**1**) is a common secondary metabolite of Cordyceps with heathy functions, and related to fungal sporulation and environmental adaption (Hu and Li 2007; He et al. 2018; Velez et al. 2007). Among the other 11 secondary metabolites there are 5 NRPs (**13** to **17**), 2 glycosides (**6** and **7**), 2 alkaloids (**11** and **12**), 2 nucleosides (**4** and **5**) and 1 terpene derivate (**8**).

NRPs (compound **13** to **17**) are existed in most cordyceps. Among them the beauveriolides (**13** and **14**) have anti-aging, beta-amyloid lowering and anti-atherogenic activities, while beauvericins (**15** and **17**) have insecticidal, induction of cell apoptosis and ionophoric property, but they have also cytotoxicity (Chen et al. 2020). N^6^-(2-Hydroxyethyl)-adenosine (**4**) was the first time detected from *C. fumosorosea*. It exists in several other cordyceps and has Ca^2+^ antagonists, anti-cancer, inotropic and renal protection activities, but it also induces oxidative stress (Kim et al. 2014; Chen et al. 2020). The known compound N^6^-(2-hydroxyethylacetate) (**5**) adenosine is an analogue of N^6^-(2-hydroxyethyl) adenosine and was identified from *C. fumosorosea* for the first time. Ovalicin-4α-alcohol (**8**) which was originally reported from *Metarhizium anisopliae* is a arterialisation inhibitor, a vascularisation inhibitor and an antipsoriatic agent (Yamaguchi and Hayashi 2010).

The compounds **6**, **7** and **11** are novel compounds and identified as fumosoroseanoside A (**6**), B (**7**) and fumosoroseain A (**11**). Bioactivity tests showed that fumosoroseanoside A and B had antibacterial and antifungal activities which may contribute to the niche competition of *C. fumosorosea* against other bacteria and fungi which would live on the killed pests. Fumosoroseain A (**11**) shows anti-aging activity, suggesting that this compound has potential to be developed as antiaging agent (Adrien et al. 2017; Gems and Partridge 2008; Vayndorf et al. 2013). Apart from the new compounds, the known secondary metabolites N^6^-(2-hydroxyethyl)-adenosine, N^6^-(2-hydroxyethylacetate)-adenosine, ovalicin-4α-alcohol, beauveriolide I, and E have also healthy or medicinal bioactivities (Hu and Li 2007; Furuya et al. 1983; Kim et al. 2014). Obviously, the metabolites of *C. fumosorosea* have pharmaceutical potentiality. This study established metabolic development basis for *C. fumosorosea*.

## Supplementary Information

**Additional file 1:** **Figure S1.** 1D NMR and 2D NMR spectrometry of Compound **5** in CD3OD. **Figure S2.** 1D NMR and 2D NMR spectrometry of Compound **6** in (CD3)2SO. **Figure S3.** ^1^H and ^13^C NMR spectrometry of Compound **7** in (CD3)2SO. **Figure S4.** 1D NMR and 2D NMR spectrometry of Compound **9** in CD3OD. **Figure S5.** 1D NMR and 2D NMR spectrometry of Compound **11** in CD3OD.

## Data Availability

The Supporting Information can be found with this article. It includes 1D and 2D NMR spectra of Compound 5 (Additional file 1: Fig. S1), 1D and 2D NMR spectra of Compound 6 (Additional file 1: Fig. S2), ^1^H and ^13^C NMR spectra of Compound 7 (Additional file 1: Fig. S3), 1D and 2D NMR spectra of Compound 9 (Additional file 1: Fig. S4), 1D and 2D NMR spectra of Compound 11 (Additional file 1: Fig. S5).

## Abbreviations

RCEF: Research Center on Entomogenous Fungi
μM: Micro mole per liter
1D: One dimension
2D: Two dimensions
NMR: Nuclear magnetic resonance
ESI: Electric spray ionization
HR: High resolution
MS: Mass spectrum
HPLC: High performance liquid chromatography
DMSO: Dimethyl sulfoxide
TOF: Time of flight
DEPT: Distortionless enhancement by polarization transfer
HMQC: Heteronuclear multiple quantum coherence
HMBC: Heteronuclear multiple-bond correlation
COSY: ^1^H-^1^H correlation spectroscopy
DNP: Dictionary of Natural Products
