# Supplementary material for: Metabolites and novel compounds with anti-microbial or antiaging activities from Cordyceps fumosorosea
Source: AMB Express. 2022 Apr 2;12:40. doi: 10.1186/s13568-022-01379-w (PMC8976864; doi:10.1186/s13568-022-01379-w)
Supplement: Supplementary file 1 — Additional file 1: Figure S1. 1D NMR and 2D NMR spectrometry of Compound 5 in CD3OD. Figure S2. 1D NMR and 2D NMR spectrometry of Compound 6 in (CD3)2SO. Figure S3. 1H and 13C NMR spectrometry of Compound 7 in (CD3)2SO. Figure S4. 1D NMR and 2D NMR spectrometry of Compound 9 in CD3OD. Figure S5. 1D NMR and 2D NMR spectrometry of Compound 11 in CD3OD. [file 13568_2022_1379_MOESM1_ESM.pdf]

Supplementary Information

Metabolites and Novel Compounds with Anti-microbial or Antiaging Activities from *Cordyceps fumosorosea*

Jie Wei[1], Xue Zhou[1], Mei Dong[1], Lufan Yang[1], Cheng Zhao[1], Ruili Lu[1]*, Guanhu Bao[2], Fenglin Hu[1]*

1. Research Center on Entomogenous Fungi, Anhui Agricultural University, Hefei, 230036, China
2. Natural Products Laboratory, State Key Laboratory of Tea Plant Biology and Utilization, Anhui Agricultural University, Hefei, Anhui Province 230036, China

FIGURE CAPTIONS

Figure S1. 1D NMR and 2D NMR spectrometry of Compound **5** in CD_3OD

Figure S2. 1D NMR and 2D NMR spectrometry of Compound **6** in $(CD_3)_2SO$

Figure S3. ^1H and ^{13}C NMR spectrometry of Compound **7** in $(CD_3)_2SO$

Figure S4. 1D NMR and 2D NMR spectrometry of Compound **9** in CD_3OD

Figure S5. 1D NMR and 2D NMR spectrometry of Compound **11** in CD_3OD

Figure S1

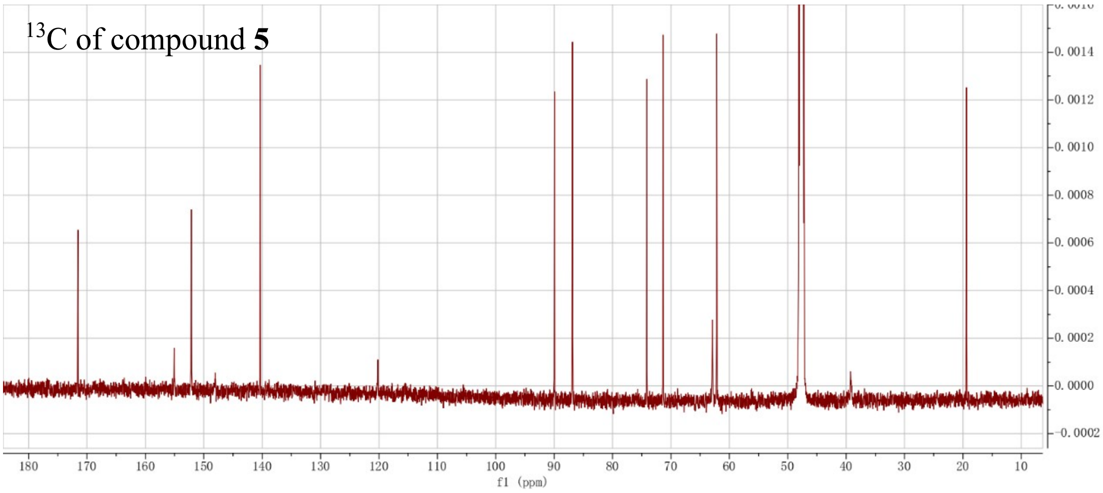

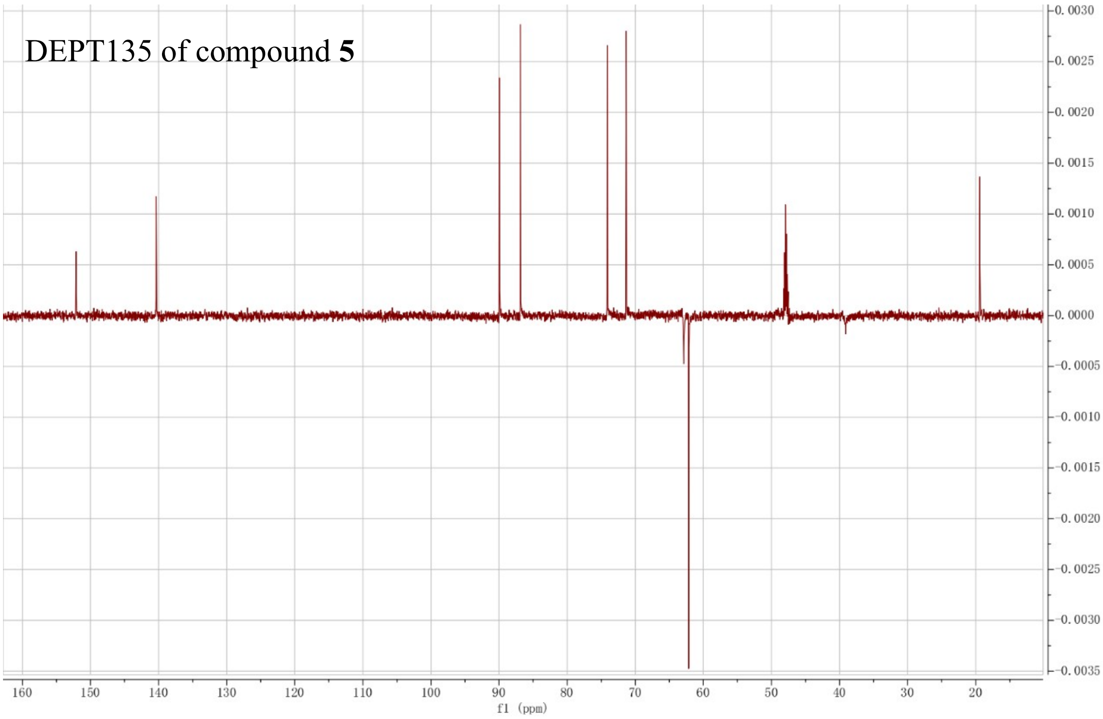

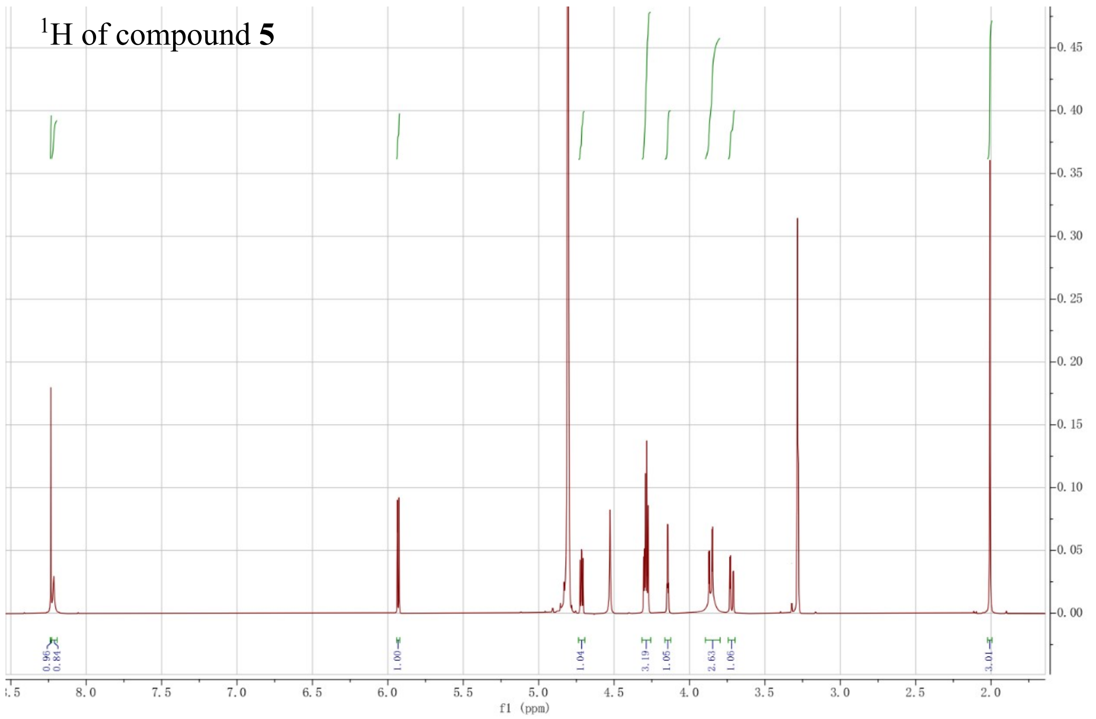

^{1}H of compound **5**
0.96
0.84
1.00
1.04
3.19
1.05
2.63
1.06
3.01
f1 (ppm)

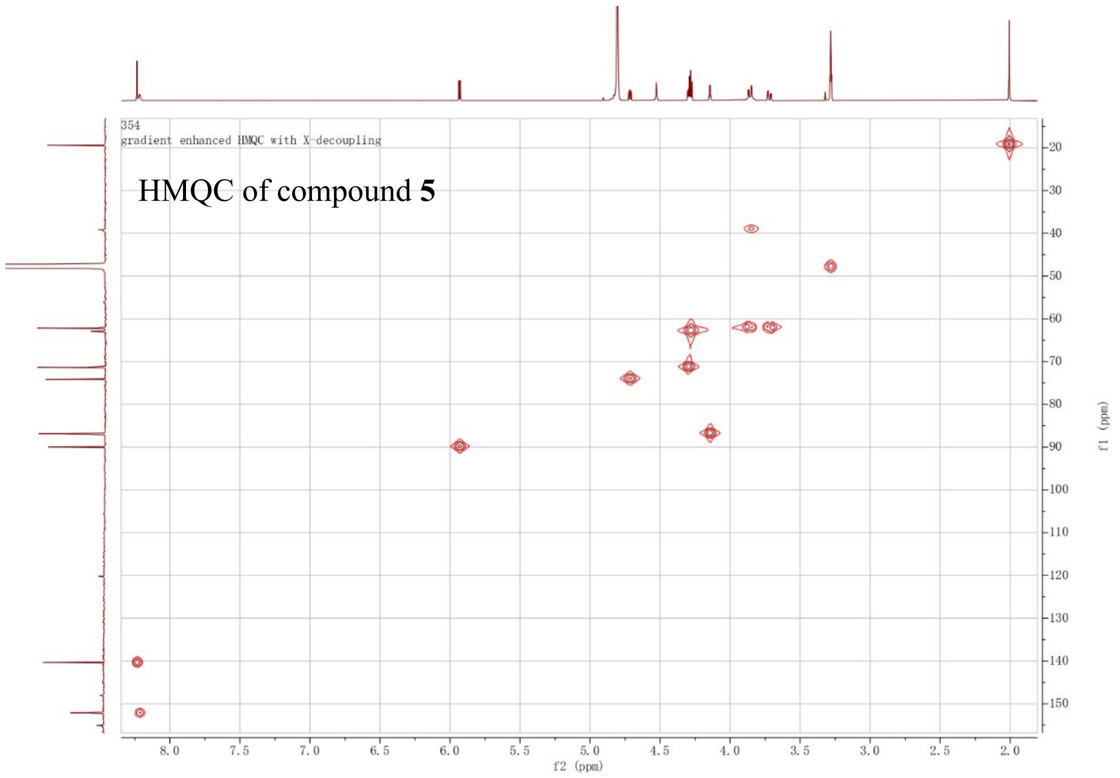

354
gradient enhanced HMQC with X-decoupling
HMQC of compound **5**
f2 (ppm)
f1 (ppm)

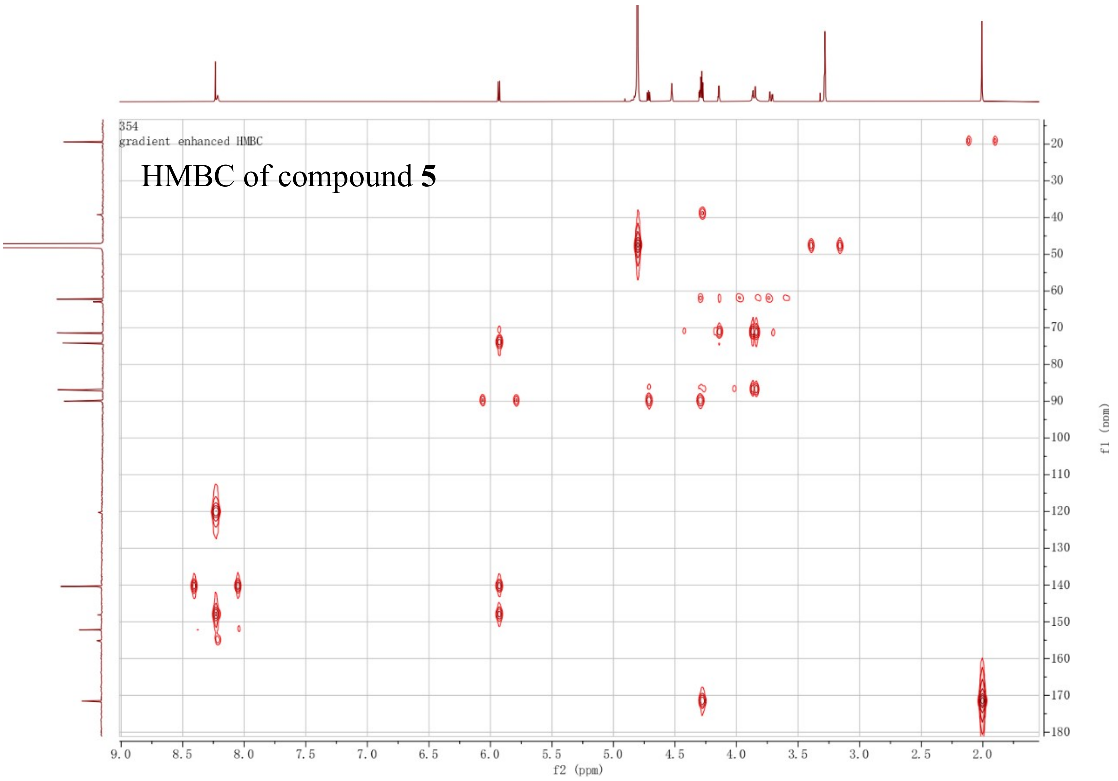
354
gradient enhanced HMBC
HMBC of compound 5
f2 (ppm)
f1 (ppm)

Figure S2

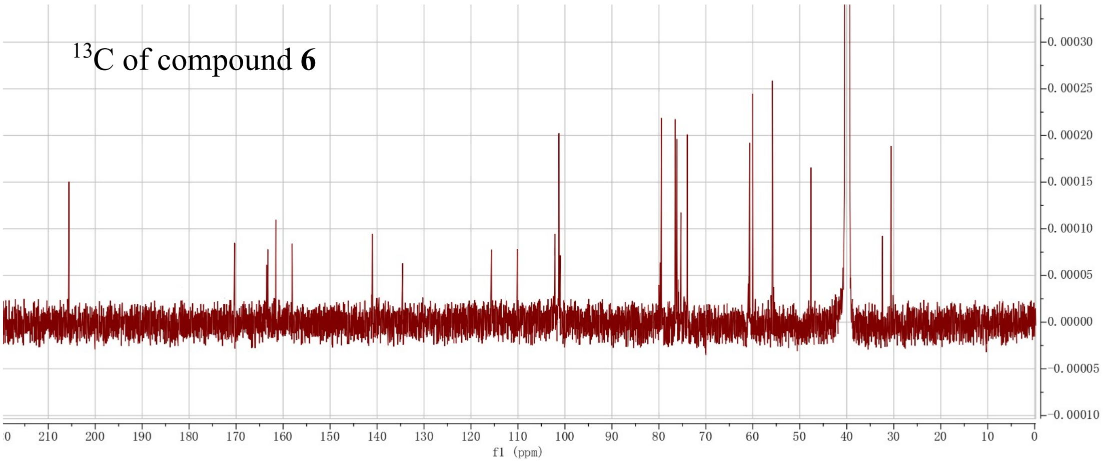
13C of compound 6
f1 (ppm)

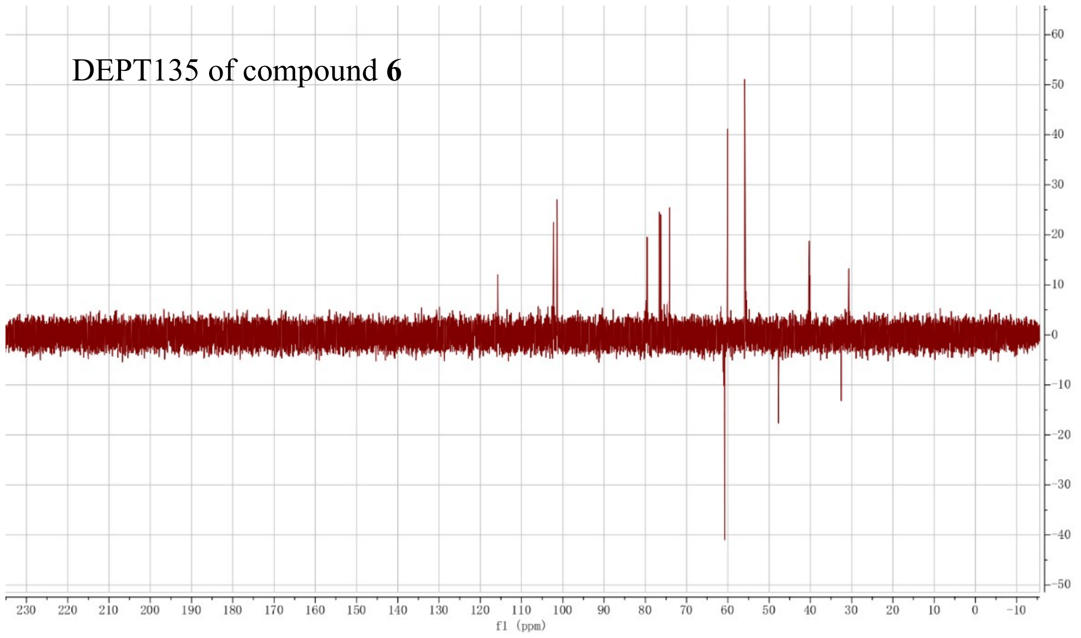
DEPT135 of compound 6
f1 (ppm)

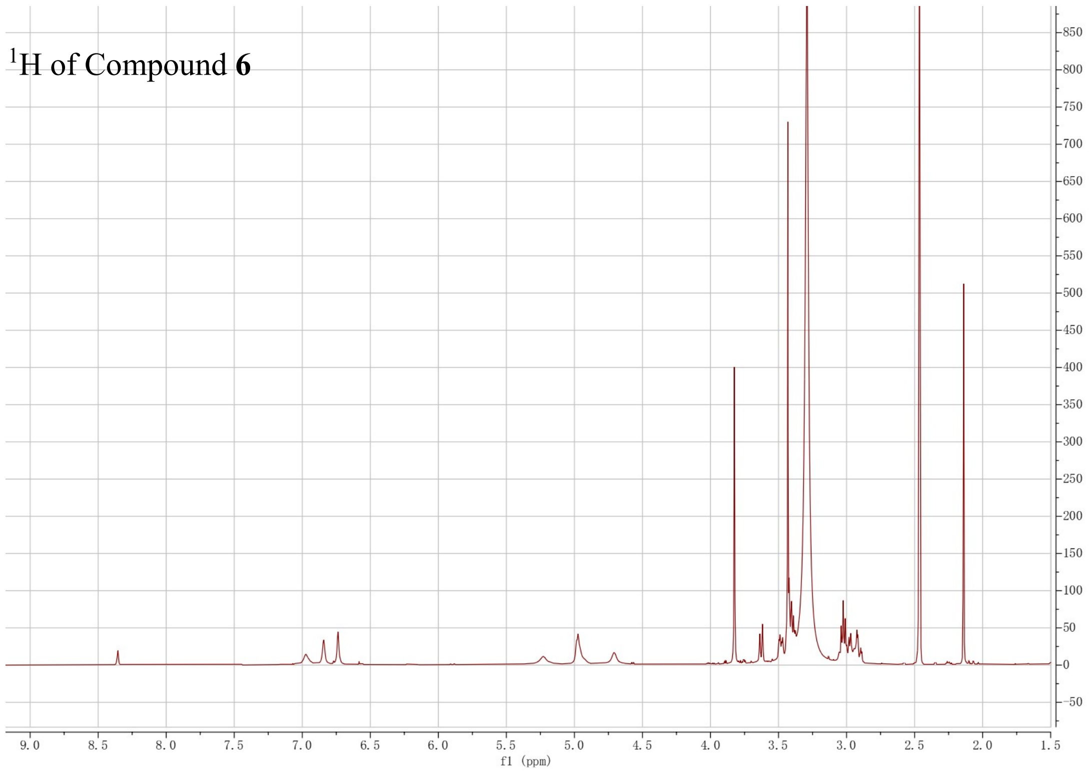
1H of Compound 6
f1 (ppm)

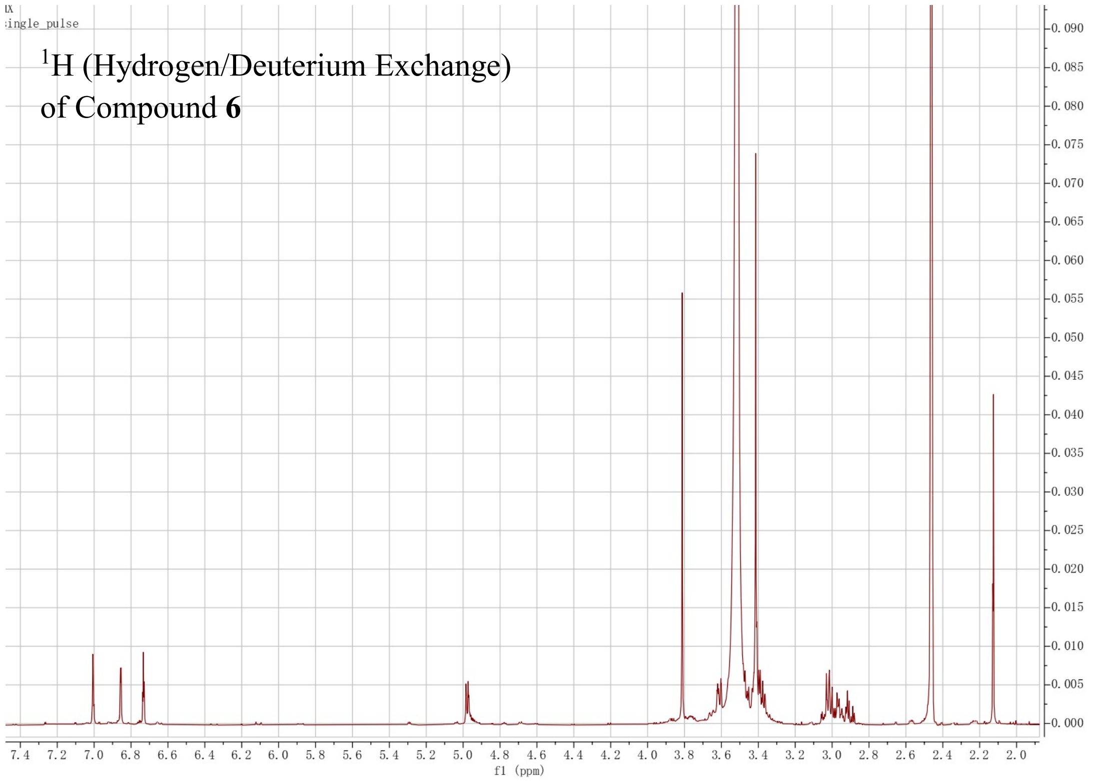

[1]H (Hydrogen/Deuterium Exchange)
of Compound **6**
f1 (ppm)

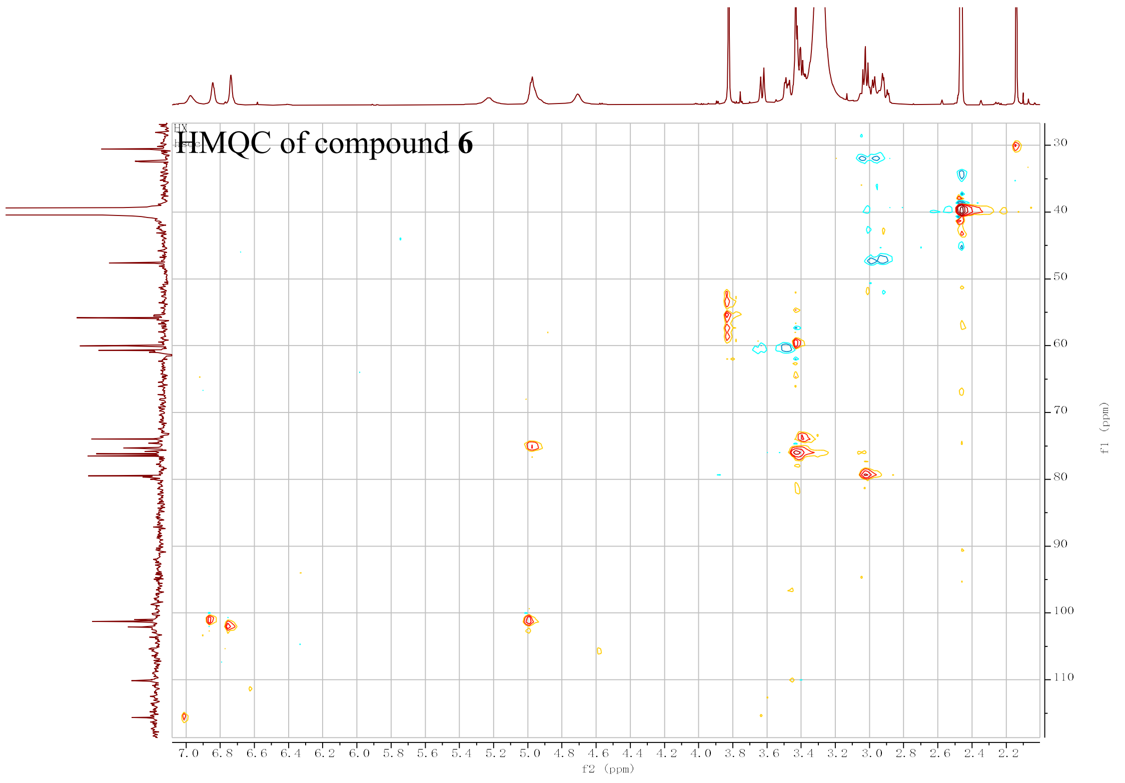

HMQC of compound **6**
f2 (ppm)
f1 (ppm)

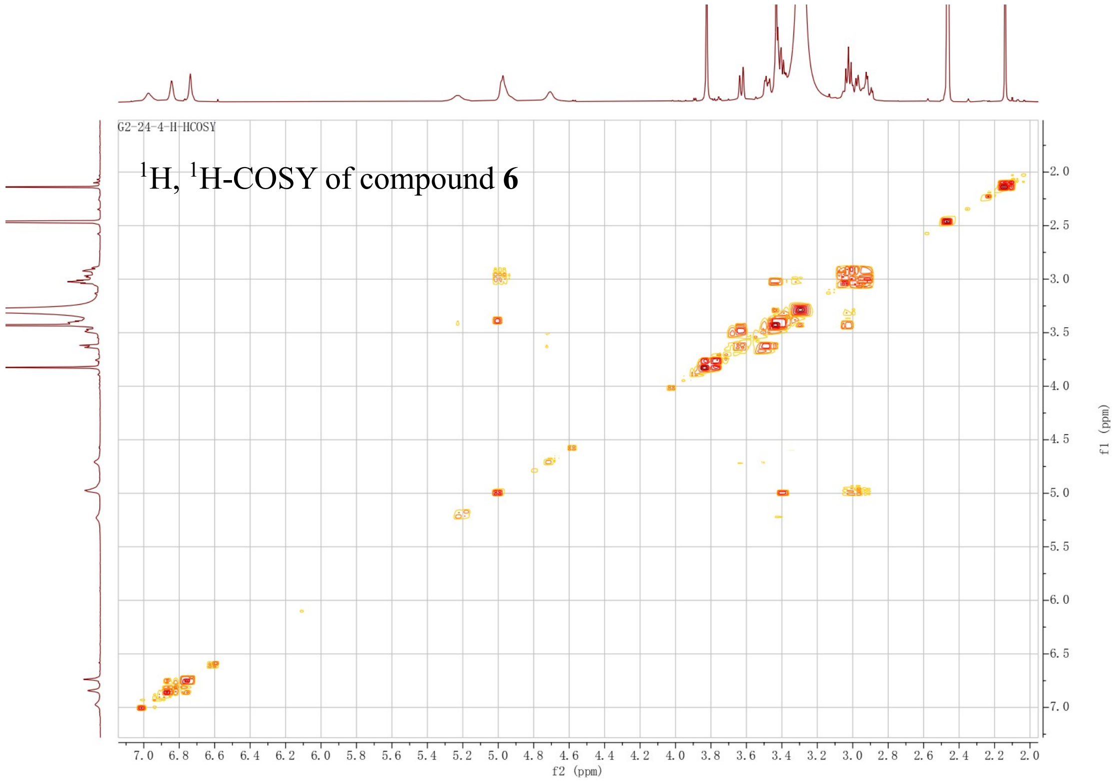
G2-24-4-H-HCOSY
^{1}H, ^{1}H-COSY of compound **6**
f2 (ppm)
f1 (ppm)

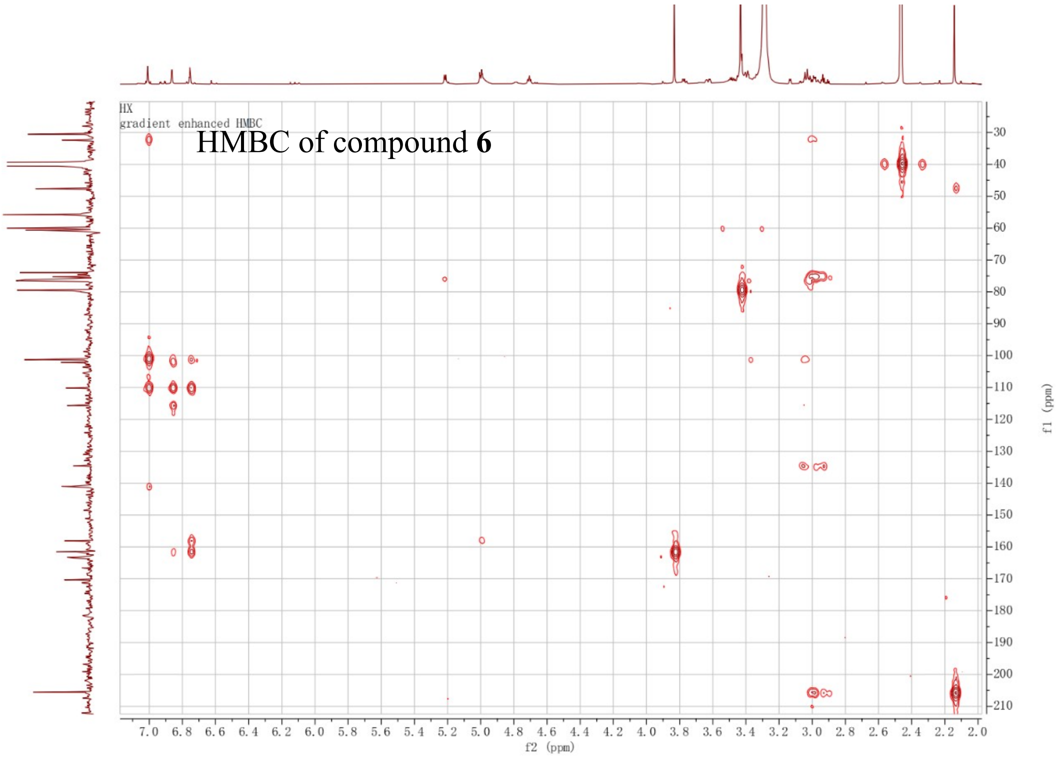
HX
gradient enhanced HMBC
HMBC of compound **6**
f2 (ppm)
f1 (ppm)

Figure S3

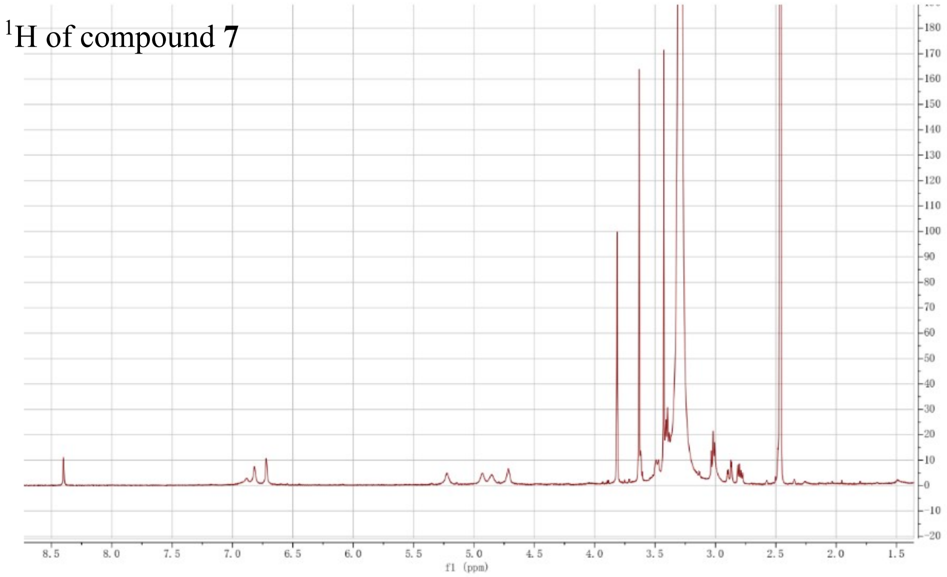

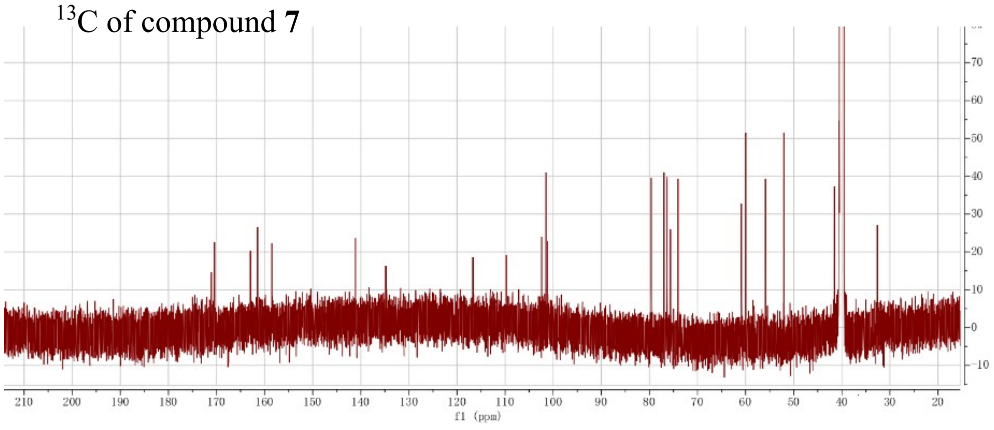

Figure S4

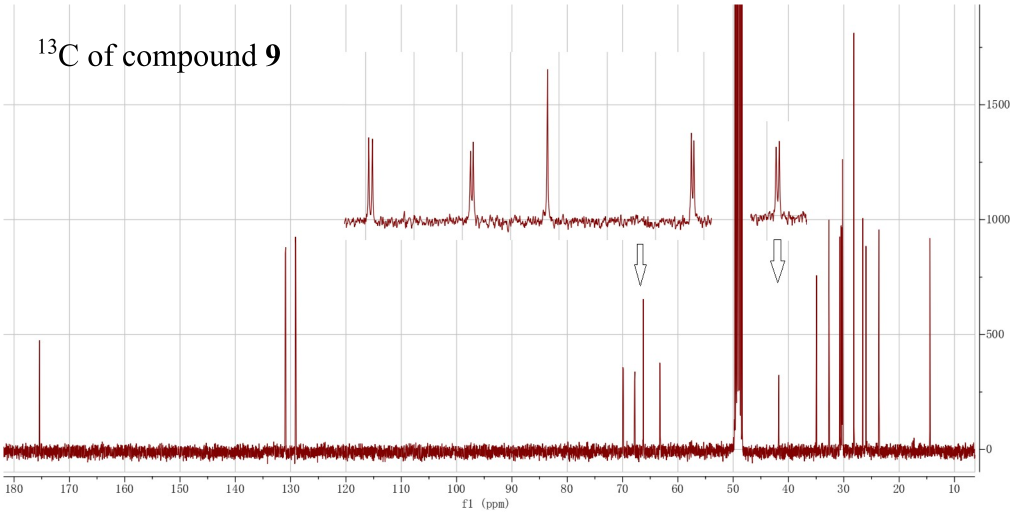

DEPT 135 of compound **9**

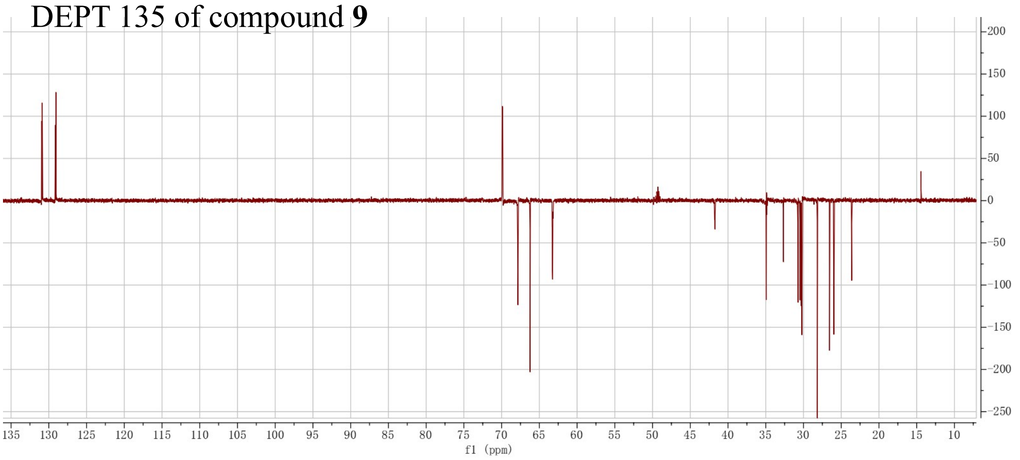

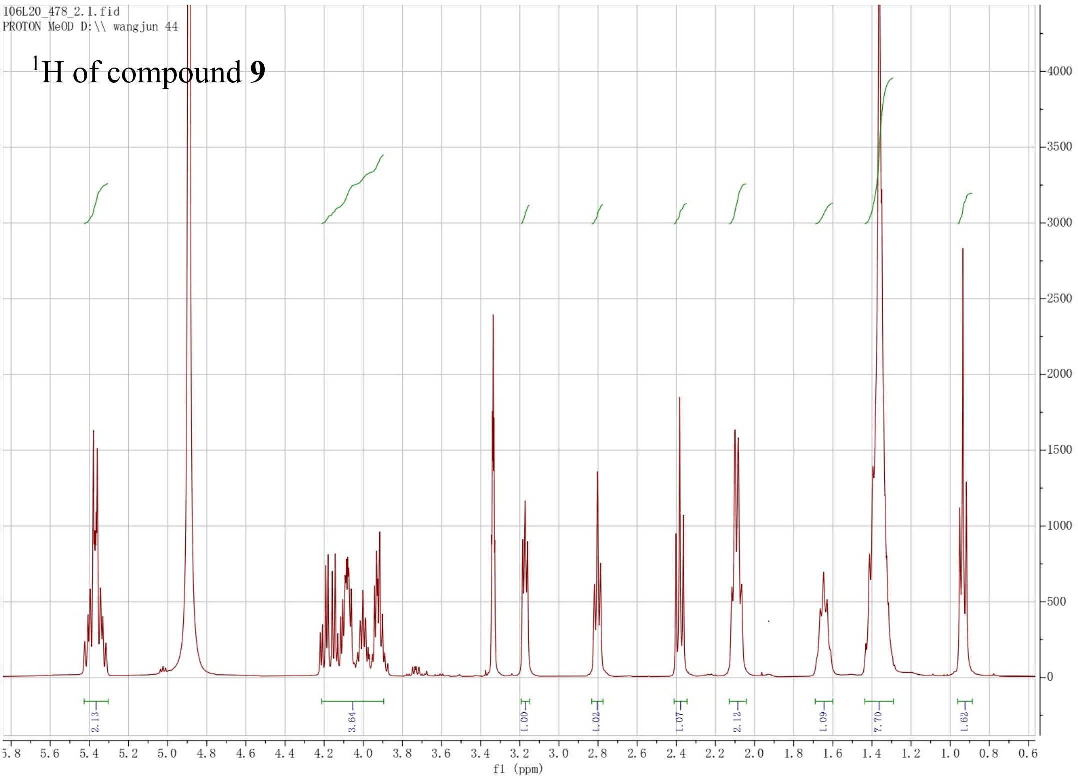

[1]H of compound **9**

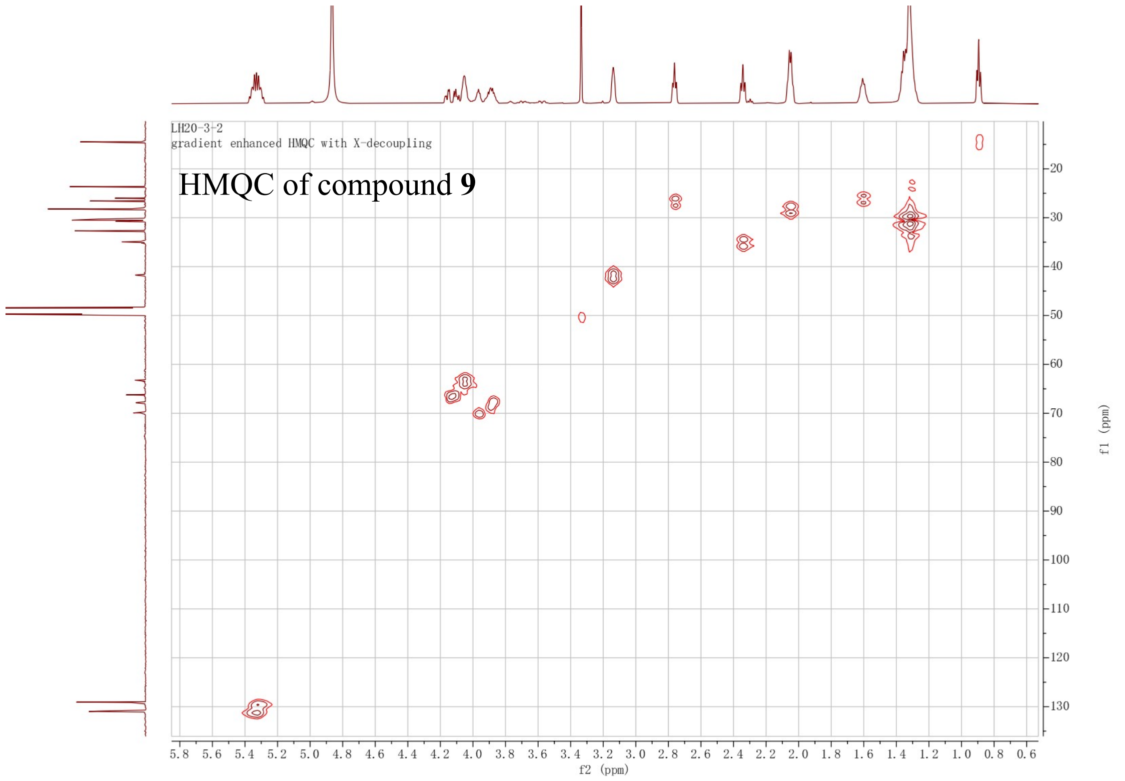
LH20-3-2
gradient enhanced HMQC with X-decoupling
HMQC of compound **9**
f2 (ppm)
f1 (ppm)

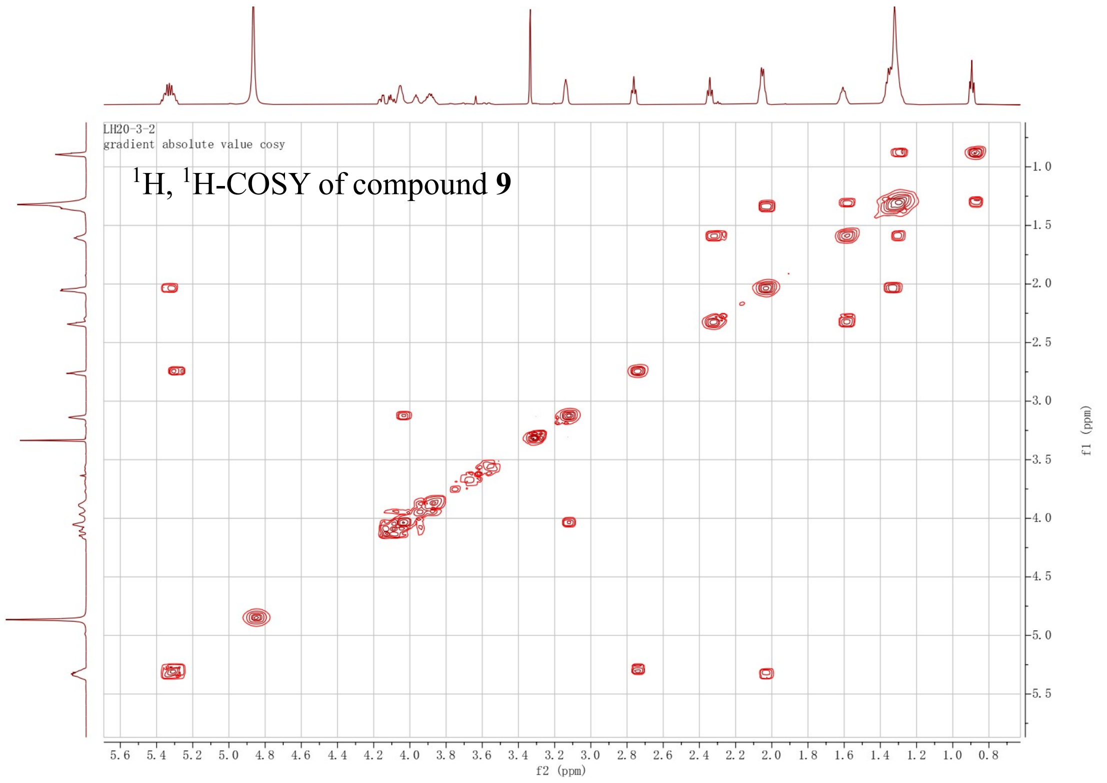
LH20-3-2
gradient absolute value cosy
^{1}H, ^{1}H-COSY of compound **9**
f2 (ppm)
f1 (ppm)

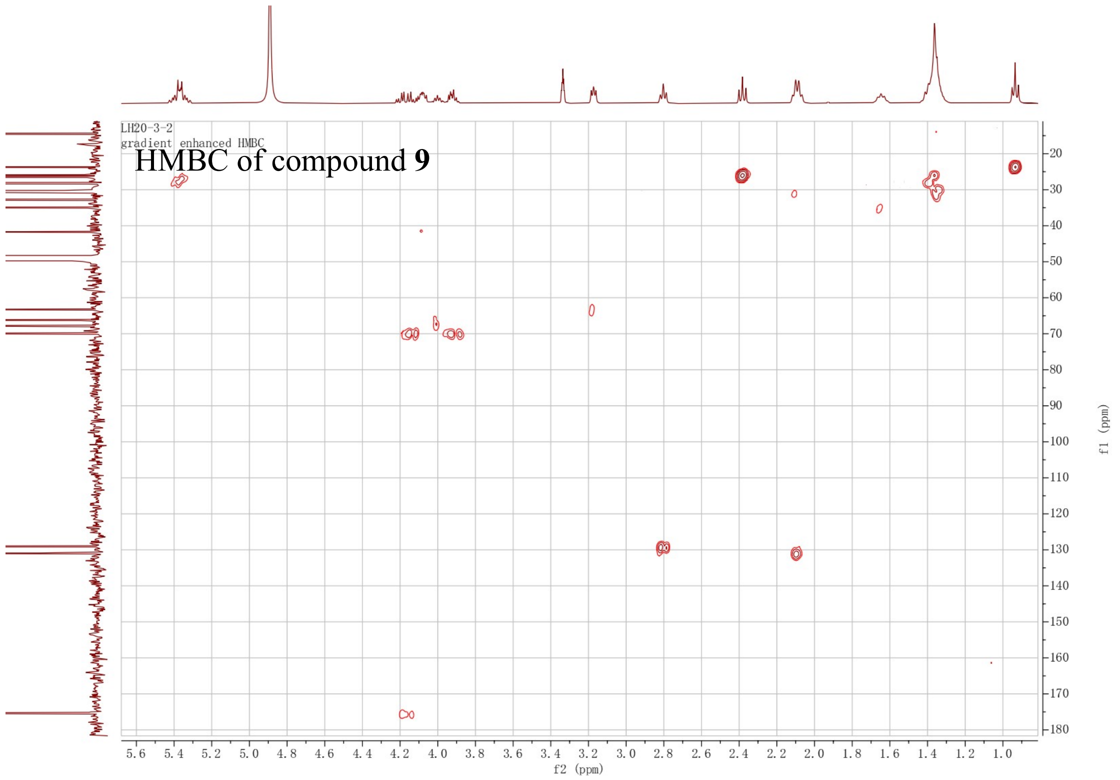
LH20-3-2
gradient enhanced HMBC
HMBC of compound **9**
f2 (ppm)
f1 (ppm)

Figure S5

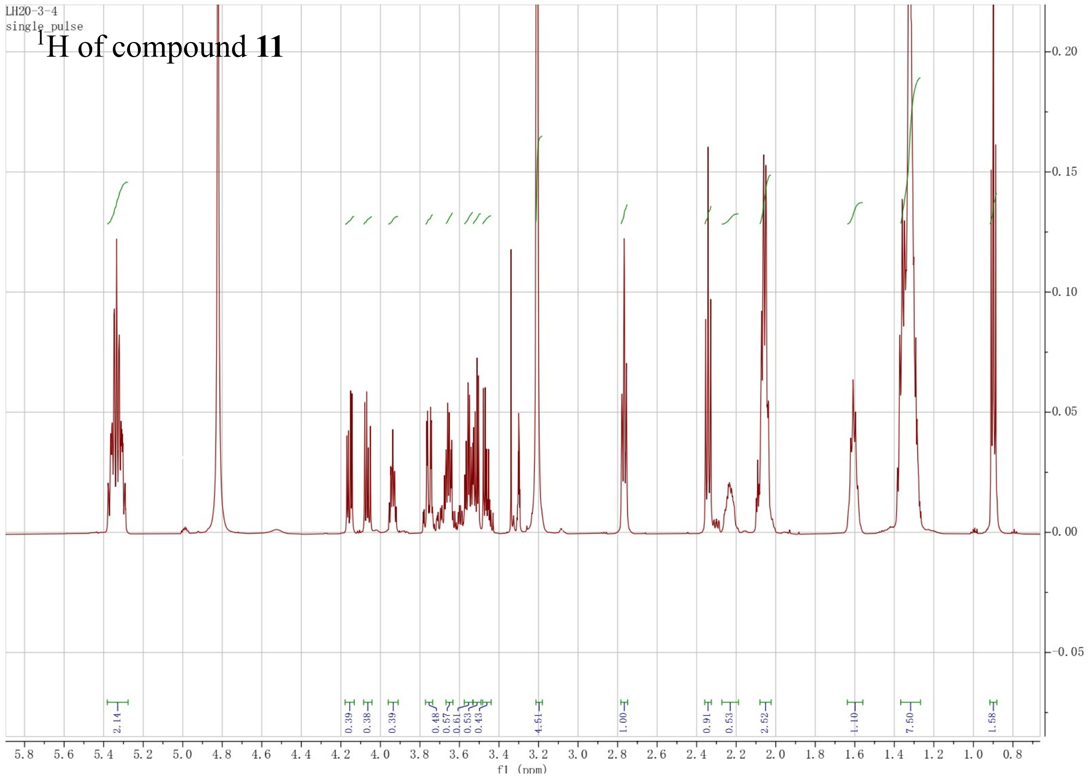
LH20-3-4
single pulse
^{1}H of compound **11**
f1 (ppm)

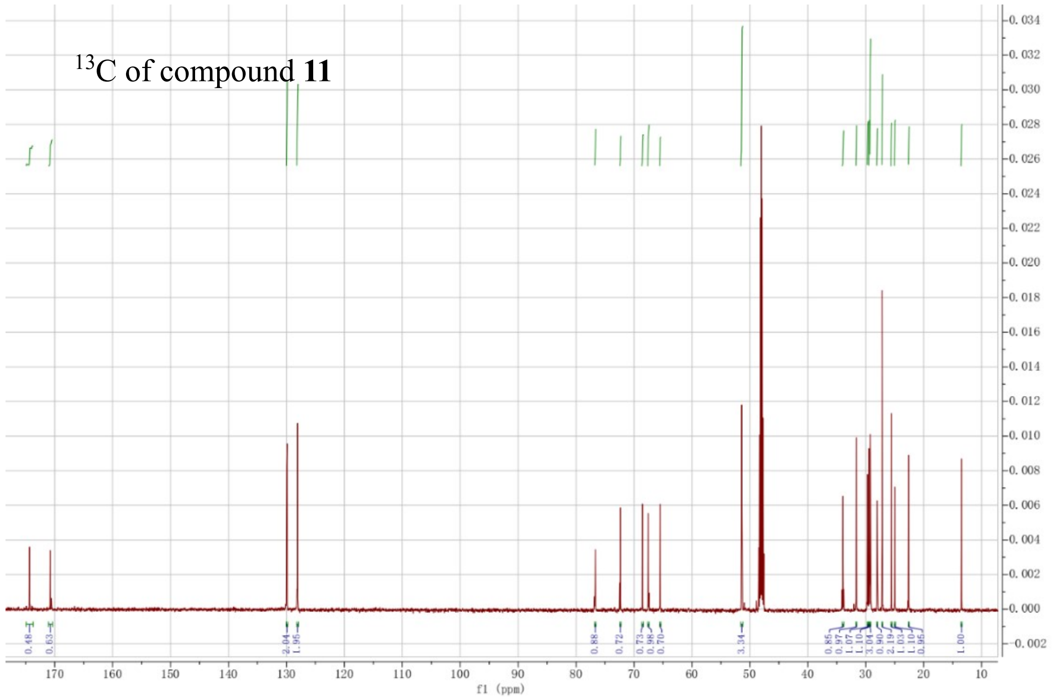
13C of compound 11
f1 (ppm)

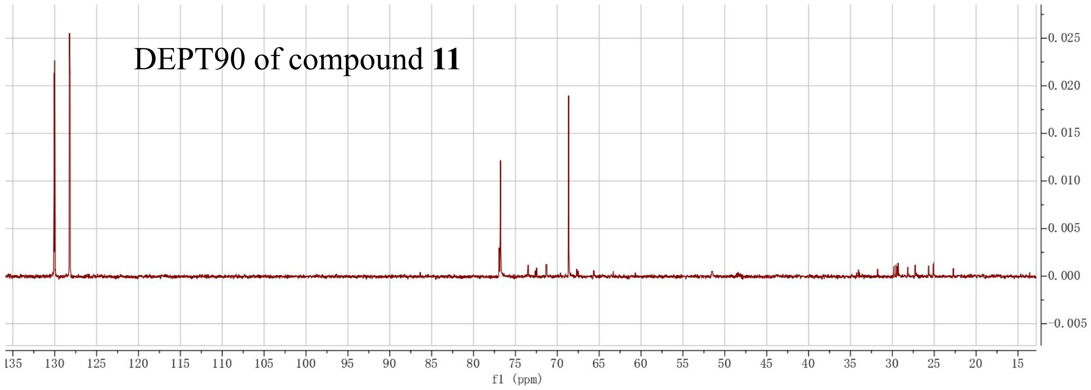
DEPT90 of compound 11
f1 (ppm)

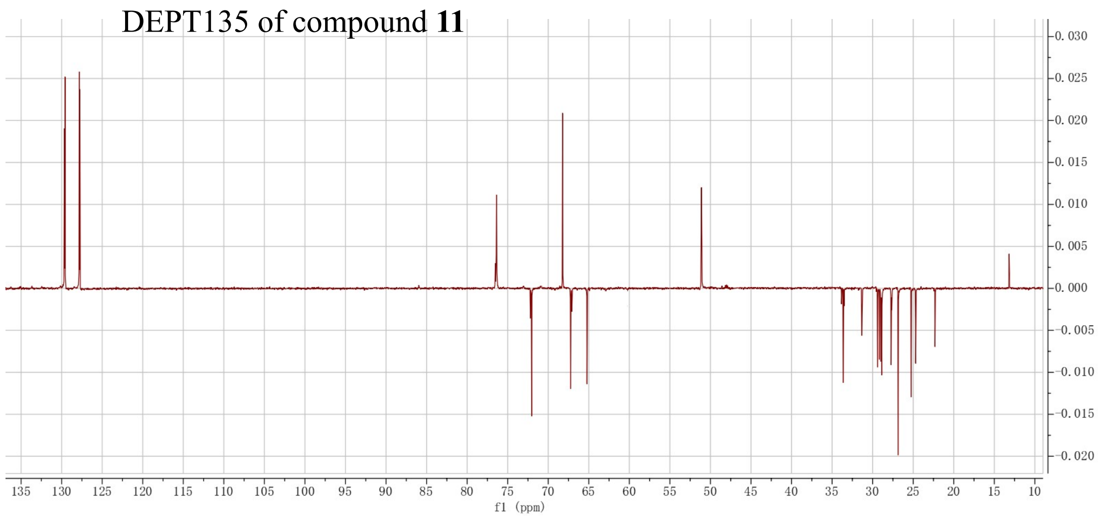
DEPT135 of compound 11
f1 (ppm)

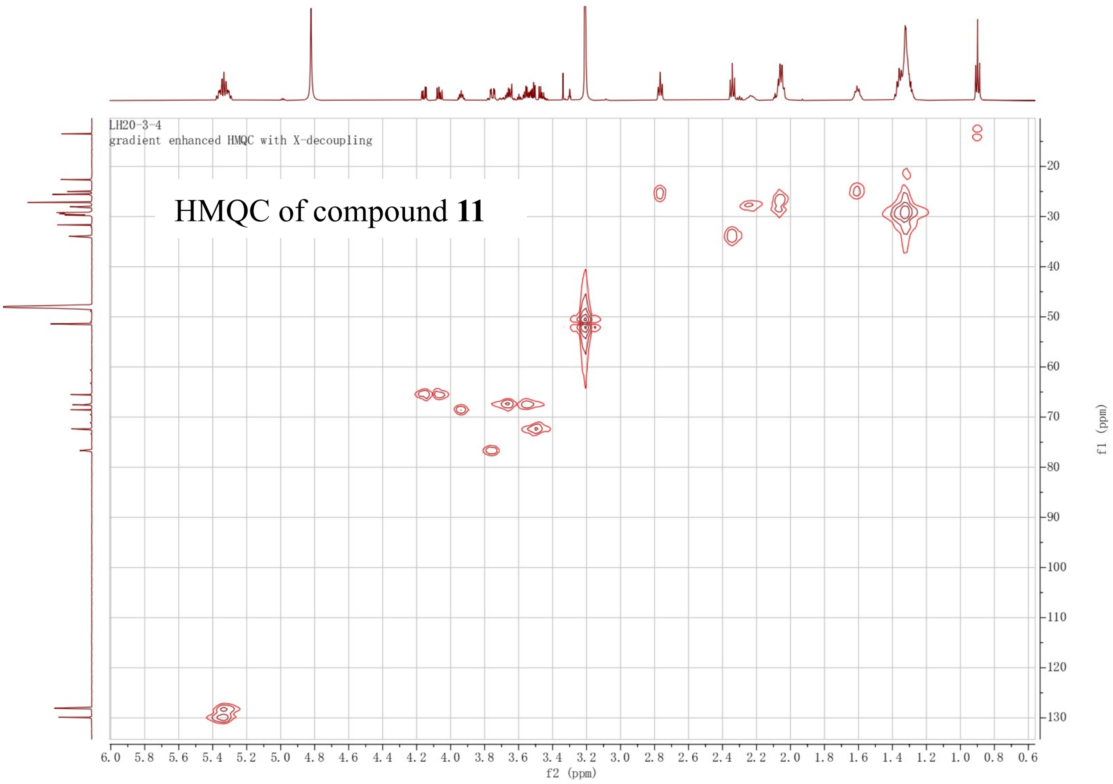
LH20-3-4
gradient enhanced HMQC with X-decoupling
HMQC of compound **11**
f2 (ppm)
f1 (ppm)

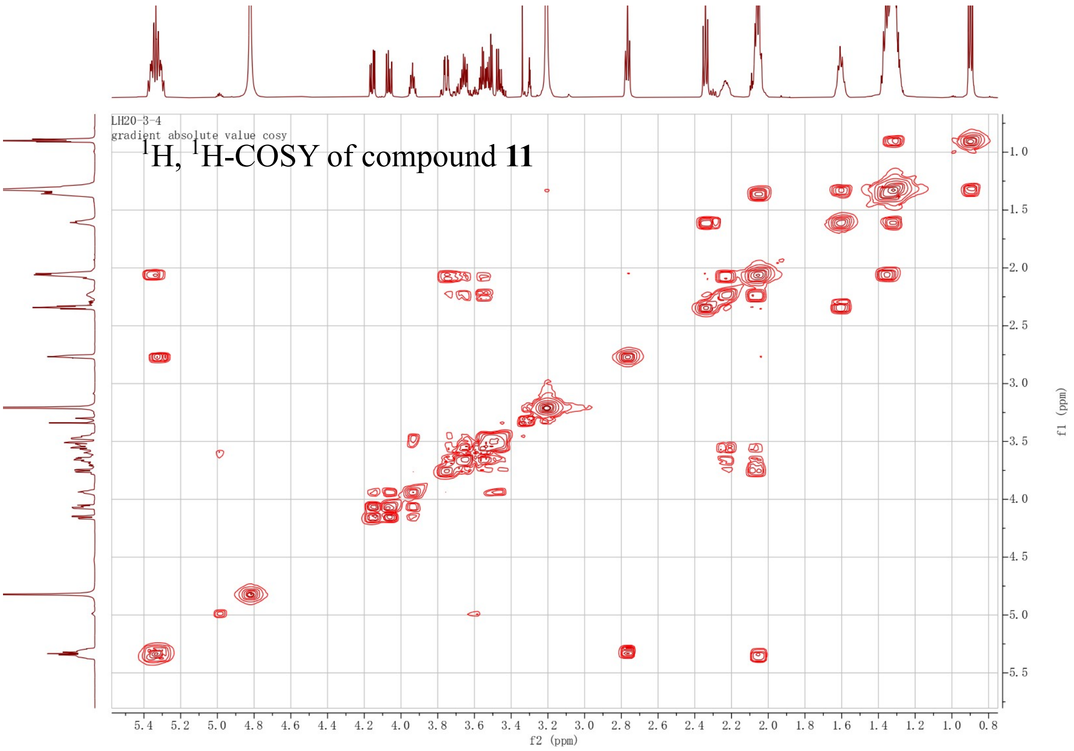
LH20-3-4
gradient absolute value cosy
^{1}H, ^{1}H-COSY of compound **11**
f2 (ppm)
f1 (ppm)

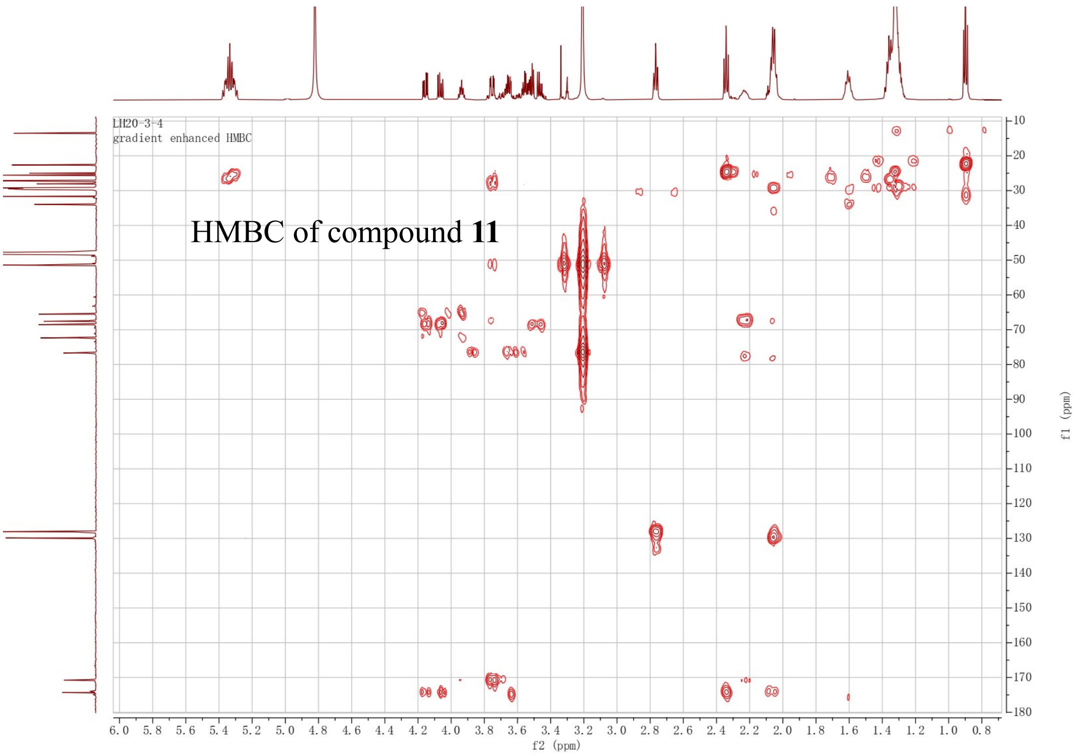
LH20-3-4
gradient enhanced HMBC
HMBC of compound **11**
f2 (ppm)
f1 (ppm)
